# Mesenchymal stem cell-derived apoptotic vesicles: emerging cell-free strategy for periodontitis treatment

**DOI:** 10.1186/s12951-025-03649-8

**Published:** 2025-10-15

**Authors:** Jingru Han, Yinyin Huang, Yadong Guo, Yongshan Li, Jiang Chen, Janak Lal Pathak, Liping Wang, Lan Yang

**Affiliations:** 1 School and Hospital of Stomatology, Guangdong Engineering Research Center of Oral Restoration and Reconstruction, Guangzhou Key Laboratory of Basic and Applied, Research of Oral Regenerative Medicine, Guangzhou Medical University, Guangzhou, 510182 China; 2 Key Laboratory of Shaanxi Province for Craniofacial Precision Medicine Research, College of Stomatology, Xi′an Jiaotong University, Xi′an, 710049 China; 3 Institute of Stomatology & Laboratory of Oral Tissue Engineering, School and Hospital of Stomatology, Fujian Medical University, 350000 Fuzhou, China

**Keywords:** Mesenchymal stem cells, Apoptotic vesicles, Immunomodulation, Tissue regeneration, Periodontitis

## Abstract

**Abstract:**

Periodontitis is a chronic infectious disease characterized by the destruction of periodontal supporting tissues. Traditional treatment methods, including scaling and root planing, have limited effectiveness in restoring damaged periodontal tissues, necessitating the exploration of new periodontitis treatment strategies. Recently, the transplantation of mesenchymal stem cells (MSCs) has made some progress in periodontitis treatment. However, scholars have found that exogenous MSCs undergo substantial apoptosis shortly after transplantation, complicating the understanding of MSCs’ specific mechanisms of action in periodontitis treatment. Notably, recent studies have reported that cells post-apoptosis can exert therapeutic effects through apoptotic vesicles (ApoVs). Several studies have confirmed that ApoVs derived from MSCs (MSC-ApoVs) have potential therapeutic effects in various disease models through immunomodulation, inflammation suppression, tissue regeneration, and drug delivery. Considering that immunomodulation, inflammatory response, and tissue regeneration are core objectives of periodontitis treatment, MSC-ApoVs show broad application prospects. This review discusses the research progress and application potential of MSC-ApoVs in the field of periodontitis treatment and compares their significant advantages over traditional stem cell transplantation techniques. This review also deepens our understanding of the mechanisms of action of MSC-ApoVs and guides us in their future clinical applications in periodontitis treatment.

**Graphical abstract:**

Illustration depicts MSC-apovs tailoring and effects MSC-apovs on functions of various cells present in periodontal tissues which indicate tremendous application potential of MSC-apovs in periodontitis treatment. Created by figdraw

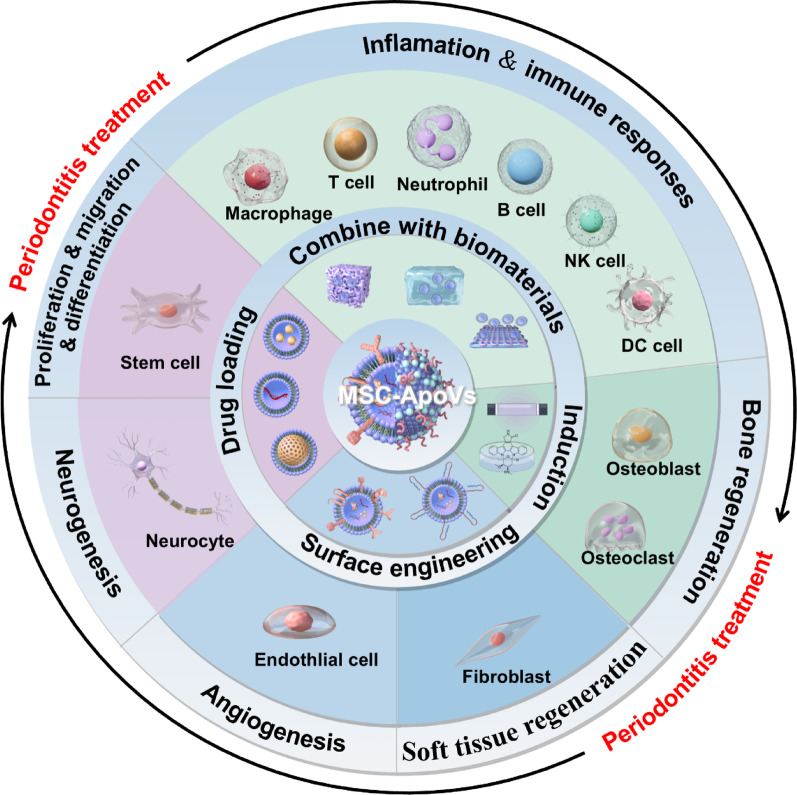

## Introduction

Periodontitis is a complex and chronic infectious disease primarily triggered by the interaction between the oral microbiome and the host immune system. The pathogenesis of this disease mainly involves the accumulation of dental plaque, which induces an inflammatory response, leading to the irreversible loss of periodontal supporting tissues, including the alveolar bone [1, 2]. The pathophysiological process of periodontitis encompasses various cellular and molecular pathways [3]. In the early stages of inflammation, the host’s innate immune system responds to the invasion of oral microorganisms by activating immune cells such as neutrophils and macrophages [4]. These cells are involved in pathogen clearance but also exacerbate the inflammatory process by releasing inflammatory cytokines like TNF-α, IL-1β, and IL-6. Neutrophils play a crucial defensive role in periodontitis, yet the reactive oxygen species (ROS) and other tissue-destructive substances they produce can exacerbate tissue damage [5, 6]. As periodontitis progresses, the adaptive immune system, including activated B and T cells, intensifies local and systemic inflammatory responses [7, 8]. Consequently, the chronic inflammatory state of periodontitis is not confined to the oral cavity but may also affect systemic health, being closely associated with diseases such as cardiovascular disease, diabetes, and rheumatoid arthritis [9–11]. Traditional periodontal treatments primarily rely on the mechanical removal of dental plaque and calculus through procedures like scaling and root planning (SRP). Although these methods effectively reduce the inflammatory symptoms of periodontal disease, they have limited effects on the recovery of already damaged tissues [12, 13]. This indicates a need for new strategies in the treatment of periodontal disease to more effectively promote the repair and regeneration of damaged tissues, aiming for a more comprehensive therapeutic outcome.

Modern periodontitis treatment strategies are gradually shifting towards tissue engineering and regenerative medicine, particularly the application of mesenchymal stem cells (MSCs), which show great potential in promoting the regeneration of damaged periodontal tissues [14–16]. The unique advantage of MSCs lies in their significant immunomodulatory properties and their ability to promote cell proliferation, migration, and differentiation, demonstrating great therapeutic potential in tissue repair, regeneration, and disease treatment [17, 18]. These stem cells can be sourced from various tissues, including bone marrow, adipose tissue, and dental pulp. In the treatment of periodontal disease, MSCs are usually injected into the damaged periodontal tissues, stimulating the proliferation and regeneration of periodontal tissue cells through their in situ regenerative capacity or by secreting growth factors and cytokines [19, 20]. Moreover, the immunomodulatory function of MSCs plays a crucial role in the treatment of periodontal disease. Studies have shown that MSCs can inhibit excessive inflammatory responses through interactions with immune cells, thereby protecting periodontal tissues from further damage [21].

However, recent studies have shown that when using MSCs to treat tissue defects, these cells undergo extensive apoptosis shortly after transplantation [22], complicating the mechanisms by which MSCs function in periodontitis treatment. Notably, multiple studies have suggested that even apoptotic cells can play significant roles [23, 24]. Apoptosis, a crucial type of programmed cell death, plays a central role in the development and homeostasis of multicellular organisms. It stimulates mitosis in surrounding cells and promotes compensatory proliferation of damaged tissues, closely related to tissue regeneration [25, 26]. Furthermore, the numerous apoptotic vesicles (ApoVs) released by apoptotic cells play a crucial role in this process. Phagocytes absorb ApoVs, facilitating intercellular signaling through the bioactive molecules they carry. Studies have shown that MSC-ApoVs significantly function in immunomodulation and in promoting cell proliferation, migration, and differentiation, demonstrating potential therapeutic effects in treatments for periodontitis, skin injuries, and osteoporosis [27, 28]. For instance, Ye et al.‘s research [29] indicated that MSC-ApoVs mitigate the macrophage inflammatory response induced by *Porphyromonas gingivalis*-derived lipopolysaccharide (LPS) and inhibit osteoclast formation. Additionally, the study demonstrated that MSC-ApoVs can effectively reduce alveolar bone loss in animal models of periodontitis, and this effect is realized by disrupting osteoclast differentiation and function. This discovery further highlights the potential therapeutic efficacy of MSC-ApoVs in the treatment of periodontitis. Therefore, this review aims to explore the research progress and application prospects of MSC-ApoVs in the treatment of periodontitis, to enhance understanding of their mechanisms of action, and to provide a scientific basis for future clinical applications.

## Periodontitis and its treatment strategies

**Fig. 1:**
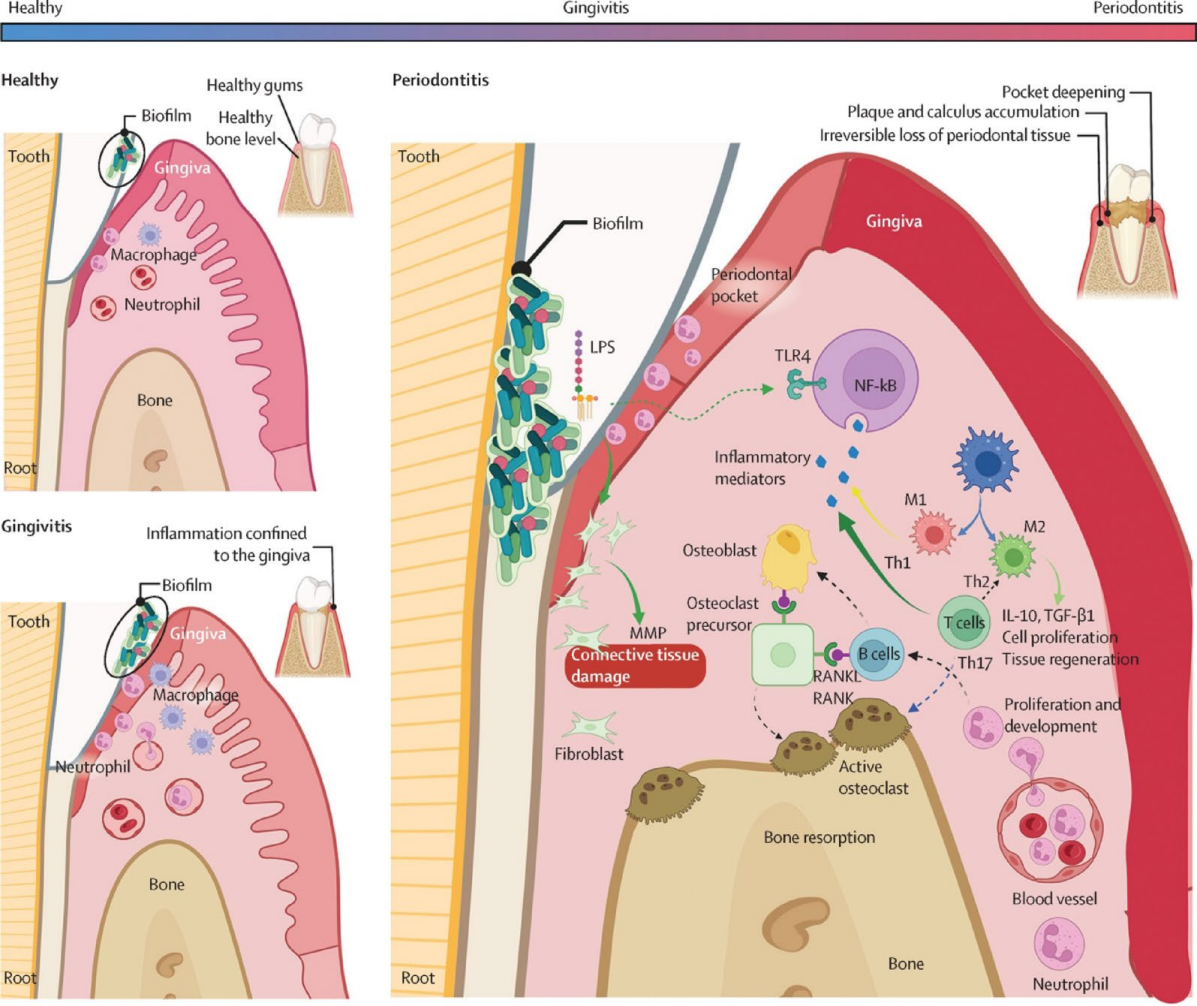
The clinicopathological progression of periodontal diseases. Reproduced with permission [31]

Periodontitis is a prevalent chronic infectious disease that primarily affects the periodontal supporting tissues, such as the gums, periodontal ligament, and alveolar bone. The progression of periodontitis involves various factors, including microbial dysbiosis, host immune responses, and the interaction between environmental and genetic factors [30, 31] **(**Fig. 1**)**. Periodontitis is usually caused by an imbalance in the oral microbiota [32]. Normally, the oral microbiota is diverse, including anaerobic bacteria, Gram-positive bacteria, and Gram-negative bacteria. However, under poor oral hygiene conditions, the number of specific pathogens such as *Porphyromonas gingivalis*, *Tannerella forsythia*, and *Treponema denticola* increases significantly [33–35]. These pathogens disrupt the microbial balance through their pathogenic LPS, triggering an inflammatory response [36]. As the number of pathogens increases, the host immune system is activated. The initial response includes the recruitment of polymorphonuclear neutrophils (PMNs) and monocytes to the inflammation site. These cells enhance the inflammatory response by phagocytosing pathogens and releasing cytokines such as IL-1β, IL-6, and TNF-α. This increases the permeability of gingival capillaries, allowing more immune cells and serum components to infiltrate the inflamed area [37]. Macrophages accumulate in the infected area, participating in bacterial clearance and exacerbating the local inflammatory response by producing prostaglandin E2 and other inflammatory factors [38]. This further promotes the recruitment and activation of immune cells, creating a self-amplifying inflammatory cycle. The chronic inflammatory environment leads to continuous tissue damage, especially due to the activation of osteoclasts, which causes alveolar bone resorption [39]. Furthermore, matrix metalloproteinases and other hydrolases produced by inflammatory cells further degrade periodontal tissues such as the gums and periodontal ligament [40]. In areas affected by periodontitis, the increased production of ROS induces oxidative stress, damaging cell membranes, DNA, and proteins. Although the host has antioxidant mechanisms to defend against these harmful substances, excessive ROS production under inflammatory conditions overwhelms these defenses, leading to cellular dysfunction and further tissue destruction [41–43]. Periodontitis not only causes tissue destruction but also disrupts tissue remodeling and repair. Normally, the balance between osteoclasts and osteoblasts maintains bone health. However, periodontitis disrupts this balance, increasing osteoclast activity and inhibiting osteoblast function, leading to alveolar bone resorption [44]. Additionally, the chronic inflammatory environment inhibits normal tissue repair mechanisms, further exacerbating periodontal tissue loss. Ultimately, this series of reactions significantly alters the periodontal tissue structure, forming periodontal pockets, increasing bacterial accumulation, damaging the periodontal ligament, causing alveolar bone resorption , and even leading to tooth loss [45]. The treatment of periodontitis relies on multiple bioactive processes, including inflammation regulation, antibacterial action, immune modulation, osteogenesis, periodontal ligament regeneration, and angiogenesis [46, 47]. Among these, SRP is a fundamental treatment that helps inhibit the progression of periodontal disease [48, 49]. However, the effectiveness of SRP is limited by the complex anatomy of tooth roots and microbial recolonization [50]. To enhance treatment outcomes, periodontal regenerative surgery is used to address bone defects caused by periodontitis, particularly in intrabony defects greater than 3 mm deep. This surgery employs various biomaterials and growth factors to promote the regeneration of cementum, periodontal ligaments, and alveolar bone while eliminating pathogens, thereby improving clinical attachment [51]. Nevertheless, the indications and efficacy of these materials remain limited, and their true regenerative potential is questioned [52]. Currently, due to the limitations of available biomaterials and growth factors, the regeneration of periodontal tissues has not yet been able to fully mimic the natural process of tissue formation [53]. MSCs are considered a potential solution due to their role in cell renewal and response to tissue injury [54].

## Application of MSCs in periodontitis treatment

MSCs are a type of multipotent stem cell capable of differentiating into various tissue types, including cartilage, bone, and muscle [55]. These cells play a key role in numerous physiological processes, such as wound healing, tissue growth, and replacing cells lost due to metabolic activities or pathological conditions [56]. MSCs can be extracted from various sources, including bone marrow, teeth, and adipose tissue. Specific types include bone marrow-derived MSCs (BMSCs), periodontal ligament stem cells (PDLSCs), and dental pulp stem cells (DPSCs) [57]. MSCs have shown significant regenerative medicine potential in the treatment of periodontal disease due to their immunomodulatory and in situ differentiation capabilities, and have been used in numerous clinical studies [58, 59]. For instance, a study by Du et al. found that the local application of BMSCs to periodontal defect sites significantly inhibits inflammation and promotes tissue regeneration, primarily relying on their differentiation ability [19, 60]. Importantly, the therapeutic effects of MSCs are not limited to their differentiation capabilities; their immunomodulatory functions are also crucial, capable of inhibiting pro-inflammatory factors, enhancing anti-inflammatory factor release, and inducing M2 macrophage polarization. Dental follicle stem cells (DFSCs) used in animal models have promoted the regeneration of the periodontal ligament and alveolar bone, while also modulating the immune response by inhibiting pro-inflammatory factors such as IL-8 and IFN-γ and enhancing IL-10 secretion [61–63]. Furthermore, reimplanted PDLSCs enhance the proliferation of anti-inflammatory Treg cells, inhibit the activity of Th1/Th2/Th17 lymphocytes, and activate the expression of ARG1, CD163, and IL-10 and suppress TNF-α secretion, thus strengthening their anti-inflammatory phenotype [64, 65].

Clinical studies have further confirmed the effectiveness of MSCs in the treatment of periodontitis. In a case reported by Aimetti et al., a 56-year-old male experienced significant regeneration in the periodontal defect area after receiving DPSCs’ transplant treatment [58, 66]. This case not only highlights the therapeutic potential of MSCs but also demonstrates their great prospects in promoting periodontal tissue regeneration and clinical applications. MSCs demonstrate significant regenerative and immunomodulatory effects in treating periodontal disease, effectively facilitating the repair and regeneration of periodontal tissues. Moreover, stem cell therapy has shown a wide range of biological actions in treating various diseases, including periodontitis [67]. However, the interactions between transplanted MSCs and the host tissue, and the underlying cellular and molecular mechanisms, are not yet fully understood. Although some studies suggest that MSCs may function by directly proliferating and differentiating at the transplantation site or by recruiting host stem cells [68, 69], recent research indicates that transplanted MSCs often undergo substantial apoptosis [70]. For instance, a study by Li et al. noted that although transplanted MSCs significantly promoted dental pulp tissue regeneration, up to 47% of the cells underwent apoptosis within 72 h post-transplantation [22]. This phenomenon introduces a new theoretical perspective that directly transplanting MSCs is not the only way to stimulate their regenerative potential; paracrine pathways may be the key solution. Existing research shows that conditioned medium from MSCs can mimic their regenerative and immunomodulatory effects in various disease models, enhancing tissue repair capabilities [69, 71, 72]. Nagata et al. further demonstrated that conditioned medium from MSCs significantly promotes the regeneration of rat alveolar bone and periodontal ligament after four weeks of transplantation by inhibiting the expression of TNF-α and IL-1β and inducing the expression of BSP-II and RUNX2 [73]. Increasing research indicates that MSCs can exert their immunomodulatory and tissue regeneration functions through paracrine pathways by secreting a plethora of cytokines and extracellular vesicles (EVs), among other bioactive molecules [58, 74, 75]. Additionally, research confirms that EVs derived from MSCs can promote the proliferation, migration, and differentiation of PDLSCs, thus facilitating the regeneration of periodontal tissues [76–78]. Although these exosomes and cytokines are primarily secreted by living cells [79], this seems hard to reconcile with how exogenous MSCs can still have positive biological effects after undergoing extensive apoptosis. However, recent research shows that MSCs, even during apoptosis, demonstrate significant therapeutic potential, especially in terms of immunomodulation and tissue regeneration [80]. Studies suggest that the release of large amounts of MSC-ApoVs during apoptosis could be the key mechanism, providing a new explanation for the therapeutic effects of exogenous MSCs despite extensive post-transplantation apoptosis.

## Characteristics of ApoVs

**Fig. 2:**
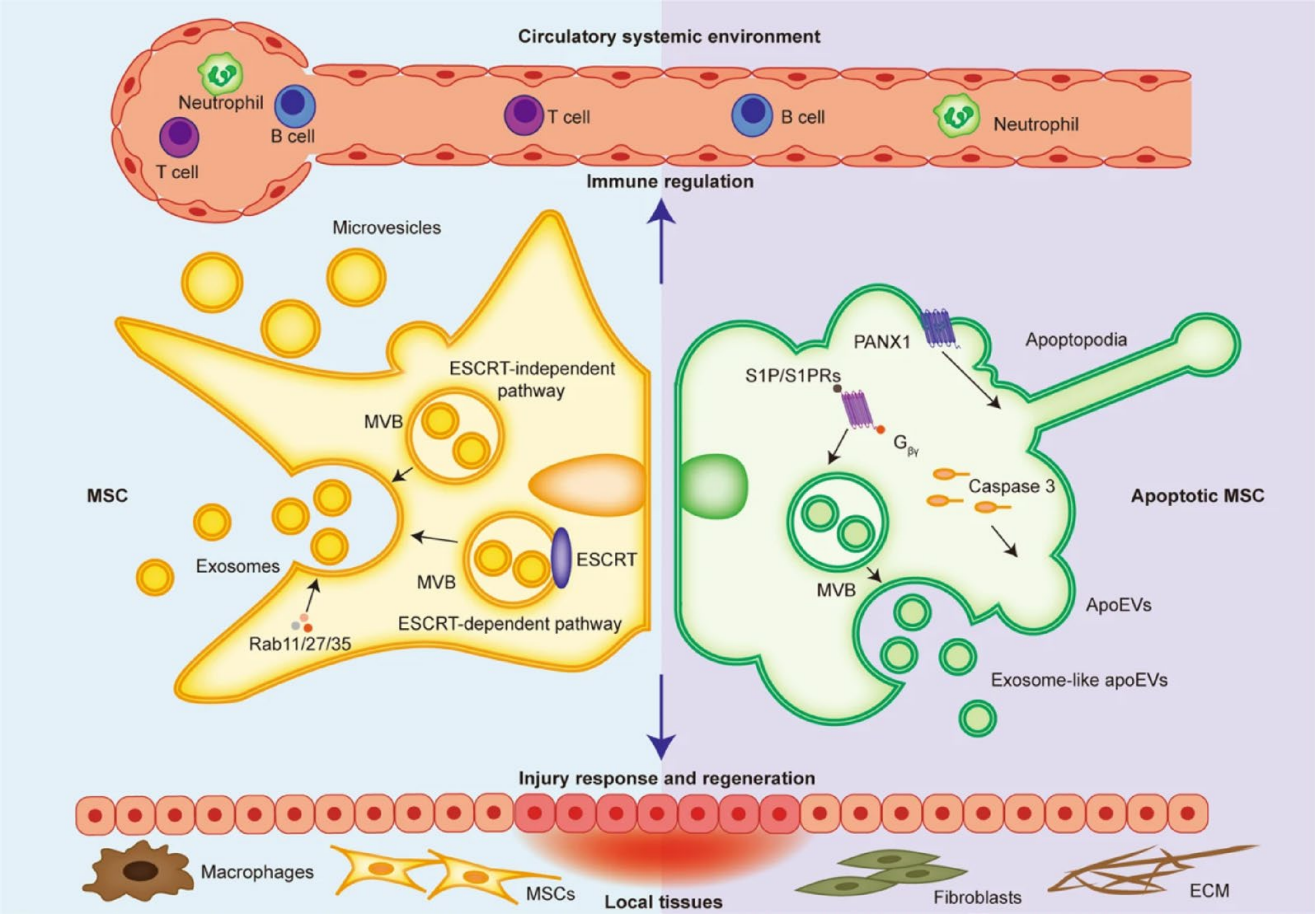
Generation and functionality of extracellular vesicles from normal and apoptotic cells. Reproduced with permission [83]

ApoVs are primarily produced by cells undergoing apoptosis, a programmed cell death essential for maintaining tissue homeostasis in organisms [81]. Unlike exosomes and microvesicles secreted by living cells, ApoVs are a unique subclass of EVs generated from cell breakdown during the final stages of apoptosis [82, 83] **(**Fig. 2**)**. They can originate from various cell types, such as progenitor cells, endothelial cells, immune cells, and MSCs [84]. The diameter of ApoVs ranges from 50 nm to 5000 nm, significantly larger than exosomes (30–150 nm) and microvesicles (50–1000 nm) [85]. During their biogenesis, ApoVs display a range of markers, including externalized phosphatidylserine (PS), calreticulin, calnexin, and BiP/GRP78 [86]. However, many of these markers are not unique to ApoVs. PS, for example, is likewise present on exosomes, microvesicles, and necrotic bodies [87]. Additionally, Zhang et al. used proteomic analysis and Western blotting to identify 13 specific biomarkers in MSC-ApoVs that distinguish them from exosomes, including Fas, integrin α−5, syntenin-4, CD44. Notably, syntenin-1 serves as a specific exclusion marker for ApoVs [88]. The formation of ApoVs begins with the condensation of chromatin within the nucleus and significant changes in cell morphology, such as membrane blebbing. These changes are driven by actin-mediated cellular contraction, leading to an increase in intracellular hydrostatic pressure [89]. Apoptotic volume decrease is a hallmark change at the early stages of apoptosis, setting the biological foundation for the formation and further development of ApoVs [90]. As apoptosis progresses, the nucleus and cytoplasm are rapidly encapsulated within multiple vesicular membranes, ultimately leading to the formation of ApoVs (Table 1).

**Table 1:** Characteristics of apoptotic vesicles, exosomes, and microvesicles

Feature	Apoptotic vesicles	Exosomes	Microvesicles
Formation mechanism	Blebbing and fragmentation of apoptotic cell membranes [82]	Released by the fusion of intracellular multivesicular bodies (MVBs) with the plasma membrane [91]	Formed by outward budding and shearing of the cell membrane [92]
Size	50–5000 nm [93]	30–150 nm [94]	50–1000 nm [95]
Secretory cells	Cells undergoing apoptosis [89]	Living cells	Living cells
Biomarkers	Caspase 3, Calreticulin, S1PR1, PS, C1q, Fas, Integrin alpha-5, Syntaxin‐4 and CD44 [96–98]	TSG101, ALIX, CD82, CD31 CD63, CD9, and CD47 [96, 99]	CD47, CD31, Annexin A1, CD9, and CD81 [100]
Content	Cellular debris, including RNAs, proteins, DNAs, lipids, and organelles [101]	RNAs (mRNAs, miRNAs, circRNAs, lncRNAs), proteins, lipid rafts, and targeting/adhesion proteins [99]	RNAs (mRNAs, miRNAs, circRNAs, lncRNAs), proteins, lipid rafts, and targeting/adhesion proteins [92]
Function in intercellular communication	Primarily involved in signaling related to cell death, inflammation, and immune response modulation [102]	Cell communication, antigen presentation, and gene regulation [94]	Cellular communication can contribute to pathophysiological conditions like tumor progression [103, 104]
Centrifugation protocols	2-Steps: 1.800 × g, 10 min, 4 °C; 2.16 000 × g, 30 min, 4 °C [86]	4-Steps: 300 × g, 10 min, 4 °C; 2 000 × g, 10 min, 4 °C; 10 000 × g, 30 min, 4 °C; 100 000 × g, 70 min, 4 °C [105]	3-Steps: 300 × g, 10 min 4 °C; 2 000 × g, 10 min 4 °C; 10 000 × g, 30 min 4 °C [105]
Yield efficiency	30 µg/10⁶ MSCs [106]	5.8 µg/10⁶ MSCs [107]	122.8 µg/1 × 10⁶ MSCs [107]
Commonality	All possess a lipid bilayer membrane and carry genetic and proteinaceous cargoes [95]		

ApoVs can be categorized into three subtypes: large micrometer-scale apoptotic bodies (ApoBDs, diameter 1–5 μm), smaller nanometer-scale apoptotic microvesicles (ApoMVs, diameter < 1000 nm), and apoptotic exosome-like vesicles (ApoExos, diameter < 150 nm). These subtypes differ not only in size but also in their functional properties [107–109]. For instance, the larger ApoBDs are likely to be more readily phagocytized by macrophages, while the smaller ApoExos may be better suited for precise intercellular communication [110]. However, due to the lack of a standardized classification, it is challenging to draw accurate conclusions about the functions of ApoVs. Therefore, we recommend that future research standardize the different subtypes of ApoVs based on their size and induction methods.

ApoVs play a crucial role in immune regulation and tissue homeostasis through specific markers and bioactive molecules on their membrane surfaces. They express membrane markers shared with exosomes and microvesicles, such as CD9, CD63, and CD81, facilitating intercellular communication [96]. In addition, PS exposed on the surface of ApoVs renders them readily detectable by Annexin V and cleared by immune cells such as macrophages, maintaining tissue homeostasis [97, 111–113]. Unlike exosomes and microvesicles, ApoVs have reduced “don’t eat me” signals like CD47 and CD31 on their surface, making them more susceptible to ingestion by phagocytic cells [114]. Moreover, the complement component C1q on the surface of ApoVs helps regulate immune responses, especially in clearing apoptotic cells and preventing excessive inflammatory responses [115, 116]. During apoptosis, calreticulin moves from the cytosol to the cell membrane and aggregates, serving as an “Eat-me” signal to enhance macrophages’ recognition and phagocytosis of ApoVs, thereby mediating their regulatory effects and potential therapeutic actions. These synergistic mechanisms not only promote the effective clearance of ApoVs but also help maintain the immune homeostasis and tissue stability [117].

The composition and functional characteristics of ApoVs are closely related to their parent cells and subtypes. Studies have shown that ApoVs can contain portions of chromatin, cytoplasm, DNA fragments, and organelles from parent cells, thereby influencing cell behavior, metabolism, transport, and regulating various diseases [88, 100, 101, 118]. Recipient cells achieve intracellular functional expression by endocytosing ApoVs containing specific proteins or miRNAs [119, 120]. For example, MSC-ApoVs can participate in maintaining bone homeostasis through the ubiquitin ligase RNF146 and miR-328-3p contained within them [121]. Additionally, different subtypes of ApoVs form at different stages of apoptosis, resulting in variations in their composition and functional characteristics [107]. Proteomic analysis shows that ApoMVs are rich in proteins related to cell adhesion, migration, and proliferation, while ApoBDs contain more molecules related to substance transport and metabolism, possibly playing a crucial role in maintaining cell homeostasis [122]. In addition, whether the parent cells are derived from young or aged individuals, or from diseased versus healthy states, can alter the cargo and functions of ApoVs [123, 124]. Therefore, future research should select appropriate parent cells and subtypes based on the expected functions of ApoVs to optimize their application effects.

**Fig. 3:**
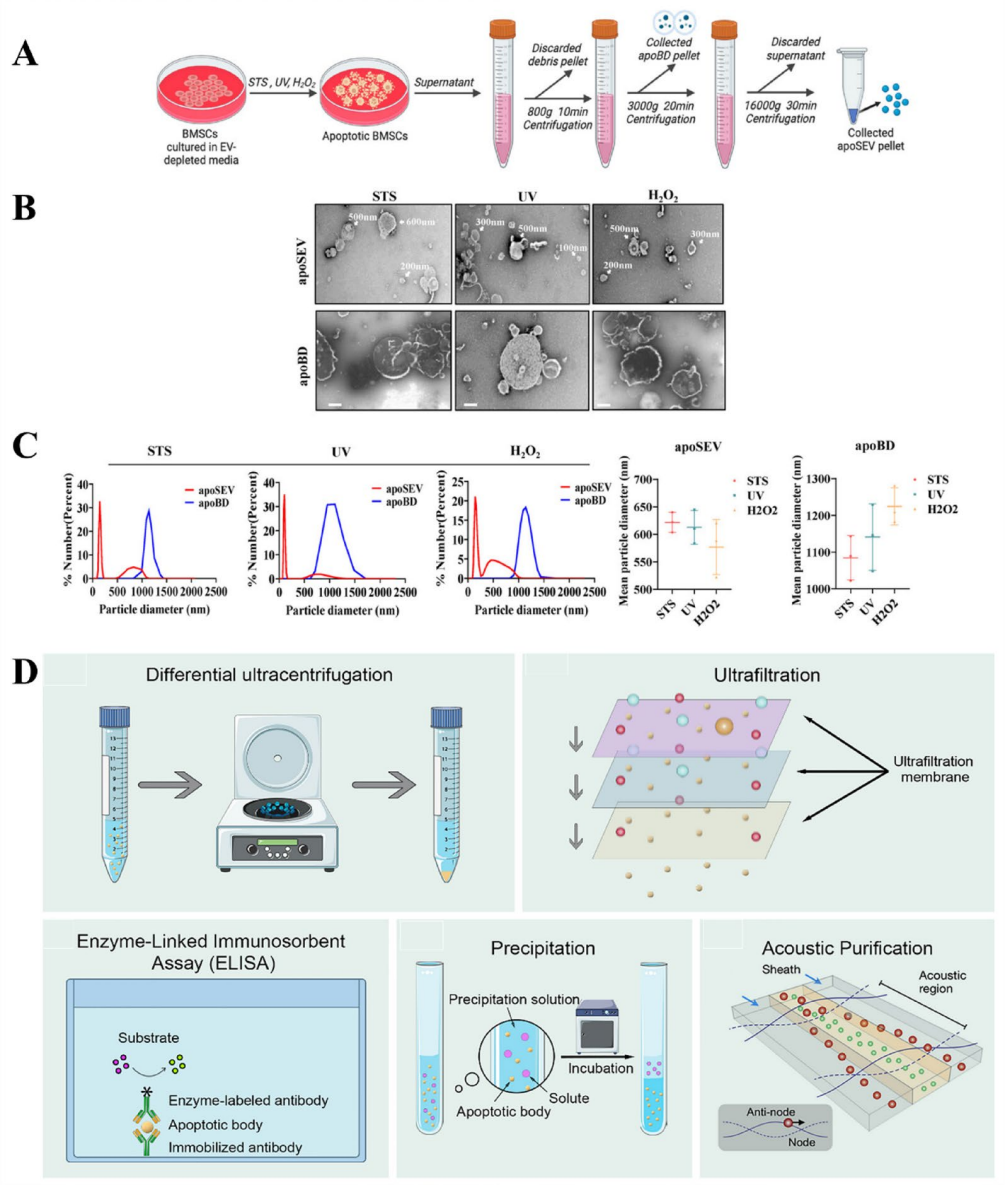
Different methods for the induction and isolation of MSC-ApoVs. (**A**) Schematic representation of the differential centrifugation method used to isolate apoptotic vesicle subtypes from apoptotic MSCs. (**B**) TEM images capturing the vesicle morphology of ApoBD and ApoSEV subtypes. Scale bar, 500 nm. (**C**) Size distribution of apoptotic vesicles assessed through DLS, demonstrating the distinct size ranges of ApoBD and ApoSEV, and their average particle size was counted. Reproduced with permission [122] (**D**) Common methods for separating and isolating ApoVs. Reproduced with permission [138]

In healthy cells, the production of ApoVs is limited, leading researchers to use various chemical and physical methods to increase cell apoptosis and thereby enhance ApoVs’ yield **(**Fig. 3A-C**)** [122]. Staurosporine (STS), a common chemical inducer, effectively induces apoptosis in MSCs within 12 h at concentrations ranging from 250 nM to 0.5 µM. STS is a broad-spectrum protein kinase inhibitor that activates caspase family proteins such as caspase-3, leading to nuclear condensation and DNA fragmentation, thus promoting the formation of ApoVs [125]. It also affects mitochondrial outer membrane permeability and promotes the release of cytochrome c by regulating Bcl-2 family proteins, a crucial step in apoptosis [126, 127]. In many studies, treated MSCs are centrifuged at 800–1000 g to remove cell debris, and then ApoVs are collected by centrifuging at 16,000 g. Biotoxicity studies indicate that thoroughly washed ApoVs contain almost no STS, ensuring their safety in experimental and clinical applications [128–130]. Other chemical agents such as hydrogen peroxide, alendronate (ALN), and the combination of serum starvation with TNF-α stimulation can also effectively induce apoptosis. Specifically, Yuan et al. reported that treating cells with 1 mmol/L H_2_O_2_ for 12 h can effectively induce apoptosis [131]. Moreover, treating cells with 500 µM ALN for 24 h can also induce apoptosis, and ApoVs can be separated by sequential centrifugation and filtration [132]. Berda-Haddad et al. used a method combining serum starvation and TNF-α stimulation to induce apoptosis. All these methods can effectively induce apoptosis [133]. Additionally, physical methods such as ultraviolet radiation and high hydrostatic pressure can also induce the formation of MSC-ApoVs. For instance, 150 mJ/cm^2^ of ultraviolet radiation can widely induce apoptosis, and 50 MPa of high hydrostatic pressure can induce apoptosis in MSCs in vitro within 36 h [101]. Hypoxic conditions can achieve similar effects; incubating MSCs in the AnaeroPack system for 72 h can induce apoptosis under hypoxia [134]. The advantage of these physical induction methods is that the cells are not exposed to any chemical agents; however, their efficiency varies compared to 0.5 µM staurosporine-induced apoptosis in MSCs [135], making staurosporine still the predominantly used ApoVs inducer. Studies have shown that different induction methods have little effect on the contents and functions of ApoVs. Whether using STS or other chemical agents such as deacetylated butyrophilin and hydroxyurea, the levels of miRNA-21-5p in induced ApoVs are significantly increased [136]. Both physical and chemical induction methods can effectively enhance cell migration and differentiation abilities [122].

In terms of ApoVs separation, besides differential centrifugation, fluorescence-activated cell sorting (FACS) is also an effective technique, although operationally complex, it can obtain high-purity samples. FACS can precisely separate ApoVs based on particle size, granularity, and the externalization of PS [137, 138]. Additionally, since ApoVs are a type of EVs, current methods used for EV separation can also be used as a reference. These methods include size-based separation (e.g., ultrafiltration), affinity-based separation (e.g., ELISA), precipitation methods, and microfluidics-based separation (e.g., acoustic purification). However, more purification methods specifically suitable for ApoVs still need to be developed [138] **(**Fig. 3D**)**. Recent research has discovered that mitochondria-rich extracellular vesicles (MitoEVs) can be isolated from EVs using specific separation methods, such as sucrose gradient centrifugation, protein-based immunoisolation, 0.2 μm filtration, and iodixanol-based high-resolution density gradient centrifugation [139–141]. This EV subpopulation, rich in diverse mitochondrial contents, can transfer mitochondrial components under various conditions, affecting the function of recipient cells and thus playing a role in the diagnosis and treatment of various diseases [142]. Mechanistically, the contents of MitoEVs change under pathological conditions, facilitating disease diagnosis [143]. Emerging evidence indicates that MSC-ApoVs can confer disease relief via mitochondrial transfer [144]. The data imply that MSC-ApoVs could serve as superior carriers of mitochondrial cargo for both diagnostics and therapy, making this avenue worthy of deeper exploration. Researchers using machine learning and bioinformatic analysis have shown that MitoEVs are highly correlated with various immune cells in periodontitis, indicating their significant potential in regulating the immune microenvironment of periodontitis [145]. However, mitochondria-rich ApoVs have not yet been studied. As larger EVs directly inherit the contents of parent cells, ApoVs have greater potential to encapsulate mitochondrial components and be used in diagnosis and therapy. This research direction is worthy of further exploration in the future.

To enhance the functionality of MSC-ApoVs, researchers have explored various preconditioning methods, including regulating the oxygen environment, cytokine activation, and 3D culture. Preconditioning simulates the complex in vivo environment to optimize the bioactivity of MSCs-ApoVs. Considering that most MSCs in the body are in a hypoxic environment (2–9% O_2_), compared to the typical laboratory setting of 21% O_2_, adjusting the oxygen concentration in culture incubators through preconditioning helps better simulate this condition [146]. Under hypoxic conditions, the number of ApoVs produced by MSCs increases, and MSC-ApoVs preconditioned in low oxygen demonstrate enhanced capabilities to promote stem cell proliferation, migration, differentiation, and superior immunomodulatory effects [147]. Furthermore, MSC-ApoVs preconditioned with oxidative stress exhibit greater angiogenic capabilities, effects primarily attributed to changes in miRNA and protein cargos within MSC-ApoVs [148]. On the other hand, cytokine activation as a pre-treatment strategy is equally effective. For example, pre-treating MSCs with IFN-γ and TGF-β1 significantly alters the bioactivity of their ApoVs, manifested by markedly inhibiting T cell proliferation, inducing the generation of regulatory T cells while maintaining their function, and reducing the number of pro-inflammatory T cells. These effects may be associated with changes in vesicle uptake efficiency by receptor cells after pre-treatment [129]. Research indicates that simulating the in vivo environment, such as adjusting the oxygen environment or using specific cytokines for pre-treatment, can significantly enhance the biological functions of MSC-ApoVs, improving their potential for application in disease treatment. The therapeutic effects of ApoVs have been studied in various diseases, and they have been proven to regulate processes such as cell proliferation, immunity, and t and repair, thus treating multiple inflammatory diseases and promoting tissue regeneration [118, 149, 150]. Previous research has found that compared to traditional 2D-cultured MSCs, 3D-cultured MSCs possess stronger stemness and proliferation abilities. This three-dimensional culture environment better simulates the in vivo cellular microenvironment, promoting cell-cell interactions and signal transmission, thereby enhancing the biological functions of stem cells [151, 152]. Additionally, 3D-cultured MSCs produce a greater quantity and higher quality of MSC-EVs, resulting in higher efficiency in promoting tissue regeneration and repair [153, 154]. Research by Zhang et al. suggests that local injection of 3D-MSC-EVs, as opposed to 2D-cultured MSC-EVs, can restore the Th17/Treg cell balance through the miR-1246/Nfat5 axis. This modulates the immune microenvironment of inflamed periodontal tissue, thereby treating periodontitis [155]. Although existing studies have preliminarily demonstrated the potential of 3D-cultured MSC-EVs in treating periodontitis, research on MSC-ApoVs produced by 3D-cultured MSCs in this field remains relatively limited. Further systematic and in-depth studies are needed to fully understand their potential biological functions and clinical applications.

**Fig. 4:**
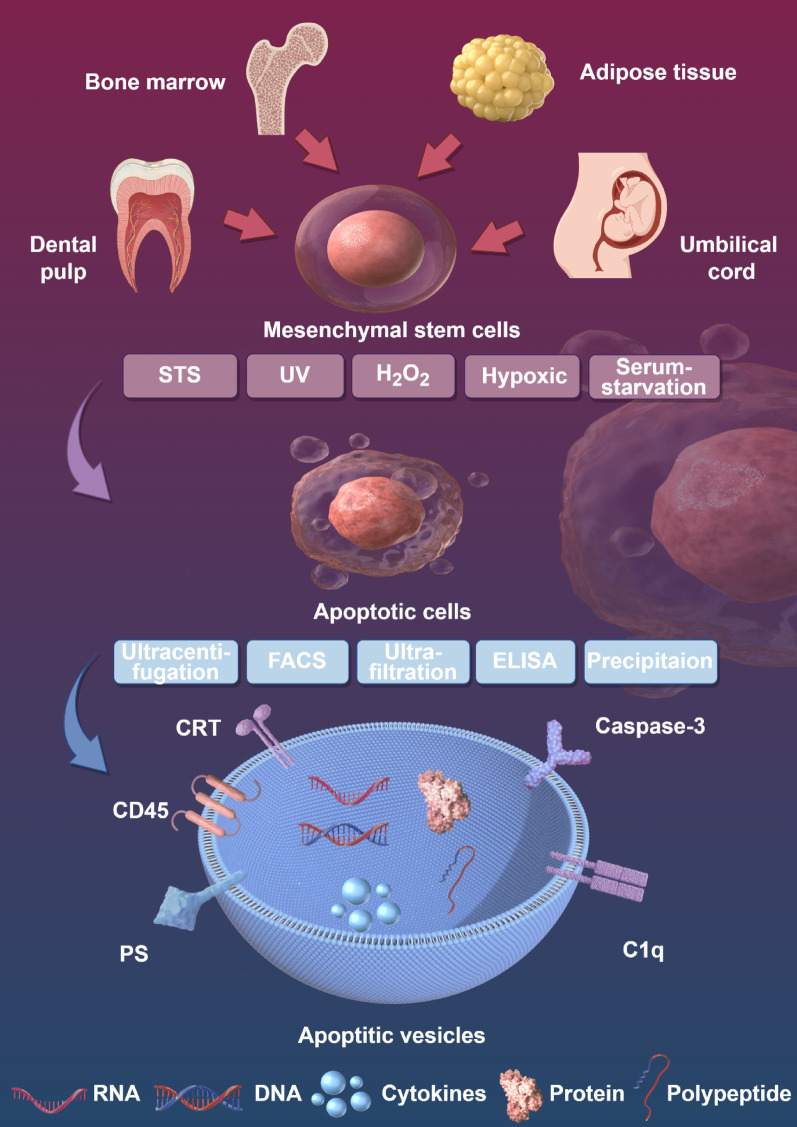
Sources, production, purification methods, contents, and surface markers of MSC-ApoVs. STS: Staurosporine; UV: Ultraviolet radiation; FACS: Fluorescence-activated cell sorting; CRT: Calreticulin; PS: Phosphatidylserine; Created by Figdraw

In summary, ApoVs are a type of EVs distinct from exosomes and microvesicles, characterized by unique surface markers and bioactive molecules that grant them specific functions. The functions and active components of different MSC-ApoVs are influenced by factors such as particle size, parent cell source, apoptosis induction methods, sorting methods, and pretreatment methods **(**Fig. 4**)**. Future research should systematically compare and optimize these parameters to determine the best MSC-ApoVs for different applications. Currently, various MSC-ApoVs have demonstrated significant effects in regulating cell proliferation, modulating immune responses, and promoting tissue regeneration and repair. This makes them suitable for treating various inflammatory diseases, including periodontitis [156–159]. The following sections will elaborate on these topics according to their respective functions.

## MSC-ApoVs and periodontitis treatment

Periodontitis is a chronic infectious disease caused by subgingival biofilm pathogens, involving various immune cells and tissue structure destruction. These pathogens activate the host’s immune response, leading to periodontal tissue damage and alveolar bone resorption, which may result in tooth loosening and even loss. Current studies show that MSC-ApoVs significantly improve periodontitis by regulating osteoclast function. However, their roles in other aspects of periodontitis, such as inflammation mitigation, immunomodulation, and tissue regeneration, remain unclear. We will explore the potential applications of MSC-ApoVs in regulating immune cell functions, supporting periodontal tissue regeneration, and serving as a drug delivery system, revealing their multiple advantages in the treatment of periodontitis **(**Table 2**)**.

**Table 2:** Function of ApoVs from different mscs’ sources and underlying mechanisms

Application	Origin	Tissue types	Induction method	Size	Target cell	Function and mechanism	Evidence level	Ref.
Immune-regulation and anti-inflammation	mBMSCs	Bone marrow	0.5 µM STS	100–1000 nm.	BMDMs	Inhibit M1 macrophage polarization via AMPK/SIRT1/NF-κB pathway	In vitro	[29]
	mBMSCs	Bone marrow	0.5 µM STS	955.41 nm(400–2000) nm	Macrophages	Promote M2 macrophage polarization	In vitro and in vivo	[118]
	mBMSCs	Bone marrow	1 µM STS	100–600 nm	Mouse splenocytes, T lymphocytes, and human PBMCs	Induce a broad spectrum of Th1/2/17 subsets while preserving the population of Treg cells via PS-mediated modulation of T cell receptor signaling	In vitro and in vivo	[160]
	hUMSCs	Umbilical cords	0.5 µM STS	100–600 nm	HEI-OC1 cells	Resist oxidative damage via FOXO3/SOD2	In vitro and in vivo	[161]
	ADSCs	Adipose	0.5 µM STS	772.45 nm (531–1110 nm)	Macrophages	Inhibit M1macrophage polarization via miR-20a-5p mediated JAK/STAT signaling	In vitro and in vivo	[162]
	hBMSCs	Bone marrow	0.5 µM STS	500 nm	PBMCs	Reduce T cell proliferation and CD69^+^ T cell activation, promotes Tregs differentiation	In vitro and in vivo	[129]
Regeneration	hUMSCs	Umbilical cords	0.5 µM STS	243.6 nm	BMSCs	Reduced hemostatic time and enhanced alveolar bone regeneration via TRIM71 upregulation and ERK1/2 pathway activation	In vitro, in vivo, and clinical	[163]
	mBMSCs	Bone marrow	Hypoxia	1 μm	Osteoclasts	Inhibit osteoclastogenesis via miR-223-3p mediated inhibition of NFIA and Itgb1	In vitro and in vivo	[134]
	hDPSCs	Dental pulp	0.25 µM STS	200 nm	BMSCs	Promote osteogenesis by activating the ERK1/2 signaling	In vitro and in vivo	[164]
	BMSCs	Bone marrow	0.5 µM STS	500 nm	BMSCs	Promote new bone formation via ROS/JNK signaling	In vitro and in vivo	[165]
	mBMSCs	Bone marrow	0.5 µM STS	100–1000 nm.	C2C12; myoblasts	Promote myoblast apoptosis via activated pannexin 1 channel-mediated creatine release	In vitro and in vivo	[128]
	mBMSCs	Bone marrow	1 µM STS	1–5 μm	mBMSCs	Promote osteogenic differentiation via ubiquitin ligase RNF146/miR-328-3p/Axin1-mediated activation of Wnt/β-catenin pathway	In vitro and in vivo	[121]
	mBMSCs	Bone marrow	0.5 µM STS	50–5000 nm	mBMSCs	Promote osteogenesis and bone formation via Ras/Raf1/Mek/Erk pathway	In vitro and in vivo	[166]
	hDPSCs	Deciduous pulp	0.5 µM STS	100–800 nm	ECs	Promote angiogenesis via the transcription factor EB-autophagy pathway	In vitro and in vivo	[22]
	rBMSCs	Bone marrow	0.5 µM STS	200–800 nm	ECs	Promote the regeneration of defective femurs by activating ER stress in ECs, which stimulates the expression of VCAM1 and recruits autologous MSCs.	In vitro and in vivo	[167]
	hSHEDs	Exfoliated deciduous teeth	0.5 µM STS	100–200 nm	ECs	Promote vascular remodeling by modulating ECs glycolysis through PD1/PDL1 axis	In vitro and in vivo	[168]
	hSNTSCs	Supernumerary tooth-derived pulp	0.5 µM STS	30–700 nm	hHUVECs	Promote angiogenesis via COL1A1/PI3K/Akt/VEGF pathway	In vitro and in vivo	[169]
	mBMSCs	Bone marrow	0.5 µM STS	955.41 nm (400–2000) nm	Fibroblasts	Promote fibroblast functions via M2 macrophage polarization.	In vitro and in vivo	[130]
	MSCs	-	0.5 µM STS	862.9 nm (745.68–908.98 nm)	ECs	Promotes cell migration via miR-210-3p-mediated AKT activation	In vitro and in vivo	[148]
	ADSCs	Adipose	0.5 µM STS	205.6 nm (100–350 nm)	BMDMs and BMSCs	Trigger M2 macrophage polarization and promote BMSCs proliferation and migration	In vitro and in vivo	[147]
	BMSCs	Bone marrow	0.5 µM STS	159.2 nm	OVX BMSCs	Salvage OVX MSCs’ osteogenic differentiation and immunoregulation via miR-145a-5p/TGF-β/Smad 2/3-Wnt/β-catenin axis.	In vitro and in vivo	[157]
	hBMSCs	Bone marrow	0.5 µM STS	100–600 nm	hBMSCs	Regulate bone metabolism via the miR-1324/SNX14/SMAD1/5 signaling	In vitro and in vivo	[170]
	hUMSCs	Umbilical cord	1 mM H_2_O_2_	100–800 nm	PC-12 cells	Reduce oxidative stress and apoptosis, and improve PC-12 cell viability	In vitro and in vivo	[171]
Drug deliver	mBMSCs (RNF146\(Asp-Ser-Ser)_6_	Bone marrow	0.5 µM STS	220–342 nm	mBMSCs	Alleviate osteoporosis via bone-targeting and promoting osteogenesis	In vitro and in vivo	[172]
	T cells (microRNA-21 or curcumin)	-	0.5 µM STS	100–600 nm	Macrophages	Ameliorate inflammatory bowel diseases by modulating cutaneous inflammation and promoting regeneration	In vitro and in vivo	[173]
	Cancer cells (vancomycin)	-	Serum Starvation	5–8 μm/80–150 nm	Raw-264.7 macrophages, U87-MG, and MDA-MB-231 cancer cell lines	Deliver vancomycin intracellularly to kill *S. aureus*	In vitro and in vivo	[174]
	hUMSCs (Cu-CDs)	Umbilical cord	0.5 µM STS	100–900 nm	BMDMs	Enhance the bactericidal activity of Kupffer cells via electrostatic binding of ApoVs with Cu-CDs	In vitro and in vivo	[175]
	hUMSCs (α-M)	Umbilical cord	5 µg/mL α-M or 1 mM H_2_O_2_	100–800 nm	BV2 microglia, BCECs, and PC-12 cells	Treat cerebral ischemia/reperfusion injury by regulating immunological response, angiogenesis, and cell proliferation	In vitro and in vivo	[171]

Note: *STS*,* Staurosporine; MSCs*,* Mesenchymal stem cells; BMSCs*,* Bone marrow-derived mesenchymal stem cells; h-*,* human; m-*,* mouse; r-*,* rat; BMDMs*,* Bone marrow-derived macrophages. SHED: human exfoliated deciduous teeth; DPSCs: Dental pulp stem cells; SNTSC: Supernumerary tooth-derived pulp stem cells; UMSCs: Umbilical cord-derived mesenchymal stem cells; OVX: ovariectomy*

### MSC-ApoVs’-mediated regulation of inflammation and immune responses

**Fig. 5:**
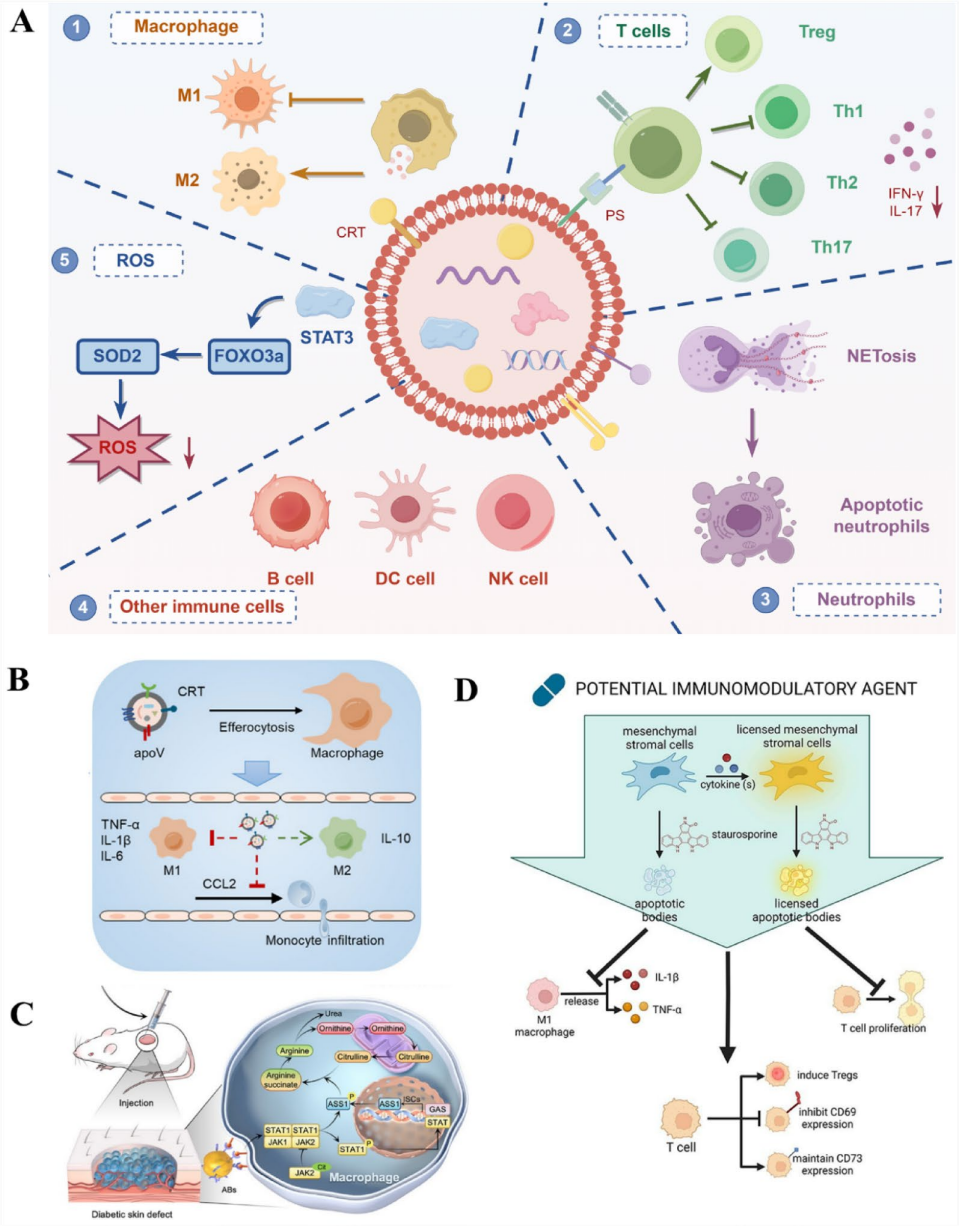
MSC-ApoVs regulate immune and inflammatory responses. (**A**) MSC-ApoVs promote M2 macrophage polarization, reduce the tendency of M1 macrophages, and improve the microenvironment of T cells through specific mechanisms. MSC-ApoVs interact with NETs by converting neutrophil NETosis to apoptosis and reduce oxidative stress responses within tissues by controlling ROS release. Additionally, MSC-ApoVs have the potential to modulate other immune cells such as B cells, DC cells, and NK cells. Created by Figdraw. (**B**) Schematic illustration shows that MSC-ApoVs modulate liver macrophage function via calreticulin (CRT) mediated efferocytosis. Reproduced with permission [118] (**C**) Schematic illustration shows the regulatory mechanism of MSC-ApoVs in balancing macrophage inflammatory polarization. Reproduced with permission [148]. (**D**) Schematic illustration shows that MSC-ApoVs have immunomodulatory effects on T cells and macrophages. Reproduced with permission [129]

MSC-ApoVs have significant immunomodulatory effects, which can influence the immune microenvironment from pro-inflammatory to anti-inflammatory by regulating different immune cells, thereby benefiting the repair and regeneration of periodontal tissues (Fig. 5A). Macrophages, key components of the immune system, play a critical role in the inflammatory response to periodontitis [176, 177]. M1 macrophages exacerbate inflammation by releasing pro-inflammatory cytokines such as TNF-α and IL-6, while M2 macrophages promote angiogenesis and bone tissue repair by producing anti-inflammatory cytokines like IL-10 and TGF-β [178]. Studies show that MSC-ApoVs can inhibit M1 polarization of macrophages, reduce TNF-α and IL-6 secretion, and increase IL-10 production via the AMPK/SIRT1/NFκB pathway, mitigating inflammation induced by *Porphyromonas gingivalis*-LPS and thus inhibiting the progression of periodontitis [29]. Furthermore, MSC-ApoVs also promote the transition of macrophages from M1 to M2 type, increasing the number of M2 macrophages and enhancing the expression of anti-inflammatory factors [118, 150, 162]. This regulatory mechanism has shown potential efficacy in treating inflammatory diseases such as periodontitis. Specifically, in treating chronic inflammation induced by type 2 diabetes and uterine lining damage, MSC-ApoVs significantly optimize the inflammatory environment and promote the repair and regeneration of damaged tissues, demonstrating their effectiveness in accelerating inflammation recovery and tissue regeneration **(**Fig. 5B, C**).**

In the periodontitis milieu, T cells play a pivotal role in immune regulation [179]. Helper T cells (Th cells), such as Th1, Th2, Th17, and regulatory T cells (Tregs), have different roles in the progression of periodontitis [180]. Research indicates that the shifts between active and inactive stages of periodontitis are closely linked to the balance of Th1 and Th2 cell immune responses [179, 181]. During the inactive phase, Th1 cells predominate, enhancing cellular immune responses by secreting IFN and IL-12, thus inhibiting bone resorption. In contrast, during the active phase, Th2 cell responses are predominant, characterized by extensive infiltration of B cells and plasma cells and a reduction in Th1 cell-associated IFN-γ and IL-12. Th17 cells promote neutrophil activity and cause tissue damage by secreting IL-17 and IL-22 [179, 182, 183], whereas Tregs maintain immune homeostasis by secreting IL-10 and TGF-β to suppress excessive immune responses [183, 184]. Research has demonstrated that MSC-ApoVs can interfere with T cell receptor (TCR) signaling via PS on their surface, directly interacting with CD4^+^ T cells to inhibit CD25 expression and IL-2 production. This action dose-dependently suppresses Th1, Th2, and Th17 effector subset activities and the production of related inflammatory cytokines such as IFNγ and IL-17, without affecting the survival of Tregs [160]. Furthermore, strategies to enhance the immunomodulatory effects of ApoVs by preconditioning MSCs with IFN-γ and TGF-β1 have shown the ability to inhibit T cell proliferation, induce Treg expansion, maintain CD73^+^ T cell activation, and reduce CD69^+^ T cell activation [129] **(**Fig. 5D**).**

Neutrophils play a crucial role in the pathogenesis of periodontitis, acting both as nonspecific pathogen-killing cells involved in immune defense and contributing to inflammation [185, 186]. During periodontitis, neutrophils function through traditional phagocytosis and oxidative burst reactions and also engage in immune defense by forming neutrophil extracellular traps (NETs) [187]. However, excessive NETs release can cause tissue damage, especially by releasing large amounts of reactive oxygen species and proteases, which disrupt periodontal tissue structure and promote bone resorption [188, 189]. Clinical studies have found that NETs levels in periodontal tissues and gingival crevicular fluid of periodontitis patients are significantly higher than in healthy controls [190, 191]. Reducing local NETs’ levels can inhibit periodontal inflammation and promote tissue regeneration. Recent research has revealed that MSC-ApoVs can interact with histones in NETs through electrostatic interactions, accumulating at sites of inflammation [192]. This phenomenon has been validated in a suppurative mouse model, indicating that MSC-ApoVs can effectively localize and exert local effects. MSC-ApoVs convert neutrophil NETosis to apoptosis via the Fas/FasL pathway, aiding in reducing excessive NETosis formation and minimizing tissue damage. This study indicates that MSC-ApoVs have considerable promise in regulating neutrophil function and thereby inhibiting periodontal inflammation.

In addition, immune cells such as B cells, natural killer (NK) cells, and dendritic cells (DCs) play crucial roles in the immune response to periodontitis. B cells differentiate into plasma cells to produce antibodies that neutralize periodontal pathogens and prevent further tissue damage [193, 194]. However, excessive activation of B cells may enhance RANKL expression, promoting osteoclastogenesis and leading to alveolar bone resorption and tissue destruction [195, 196]. NK cells recognize and kill infected or mutated cells through the release of cytokines such as interferon-γ and direct cytotoxicity. While their pro-inflammatory action helps combat infections, excessive activation may exacerbate inflammation, leading to tissue damage [197]. DCs act as a bridge in the immune response to periodontitis by presenting antigens to activate T cells, initiating specific immune responses, and interacting with other immune cells to regulate inflammation [198]. Studies have shown that apoptotic cells regulate the functions of various immune cells by releasing signaling molecules, affecting inflammation and tissue regeneration [199]. Apoptotic cells expose “eat-me” signals such as phosphatidylserine to attract phagocytes to clear them, helping to maintain tissue homeostasis and immune system balance [200]. After DCs take up apoptotic cells, they activate T cells through antigen presentation mechanisms and induce B cells to differentiate into regulatory B cells, which secrete anti-inflammatory cytokines such as IL-10, inhibiting excessive immune responses, reducing inflammation, and minimizing tissue damage [201–203]. Although there is no direct evidence that ApoVs can regulate the above immune cells, existing evidence indirectly supports this possibility. Additionally, evidence suggests that MSC-ApoVs promote the secretion of anti-inflammatory factors by various immune cells, reducing the expression of inflammatory cytokines. The inflammatory factors in the overall immune environment regulate multiple immune cells, thus playing an important role in the immune microenvironment of periodontitis [118, 148, 150, 204].

Recently, oxidative stress damage caused by excessive ROS in periodontal tissues has received widespread attention [205, 206]. During periodontitis, ROS generates cytotoxicity by directly oxidizing lipids, proteins, and DNA in cells and their extracellular matrix, or indirectly stimulating inflammatory responses and damaging the immune system, thus harming periodontal tissues [207, 208]. MSC-ApoVs have shown potential in mitigating oxidative stress damage, especially in enhancing cellular resistance to ROS. Studies have indicated that cochlear hair cells, upon uptake MSC-ApoVs, can activate FOXO3a nuclear translocation and SOD2 expression, enhancing resistance to oxidative damage, reducing hair cell loss, and effectively alleviating noise-induced hearing loss. These effects may be associated with intravesicular signaling and activation of STAT3 [161]. Oxidative stress can promote the release of various inflammatory cytokines by immune cells, exacerbating inflammation. However, MSC-ApoVs can effectively suppress the expression of these inflammatory factors such as IL-6, IL-1β, IFN-γ, and TNF-α, thereby alleviating inflammation [150]. This role underscores the potential application of MSC-ApoVs in treating periodontal diseases by resisting oxidative stress and regulating inflammatory responses. Although research in this area is not yet extensive, existing literature indicates that MSC-ApoVs play a significant role in treating inflammatory diseases. The imbalance in the immune microenvironment and excessive production of ROS are key to tissue destruction caused by periodontitis. Thus, ApoVs may have potential therapeutic benefits in regulating immune cells and the oxidative environment, thereby mitigating tissue damage and alleviating the progression of periodontitis.

### MSC-ApoVs-mediated periodontal tissue regeneration

**Fig. 6:**
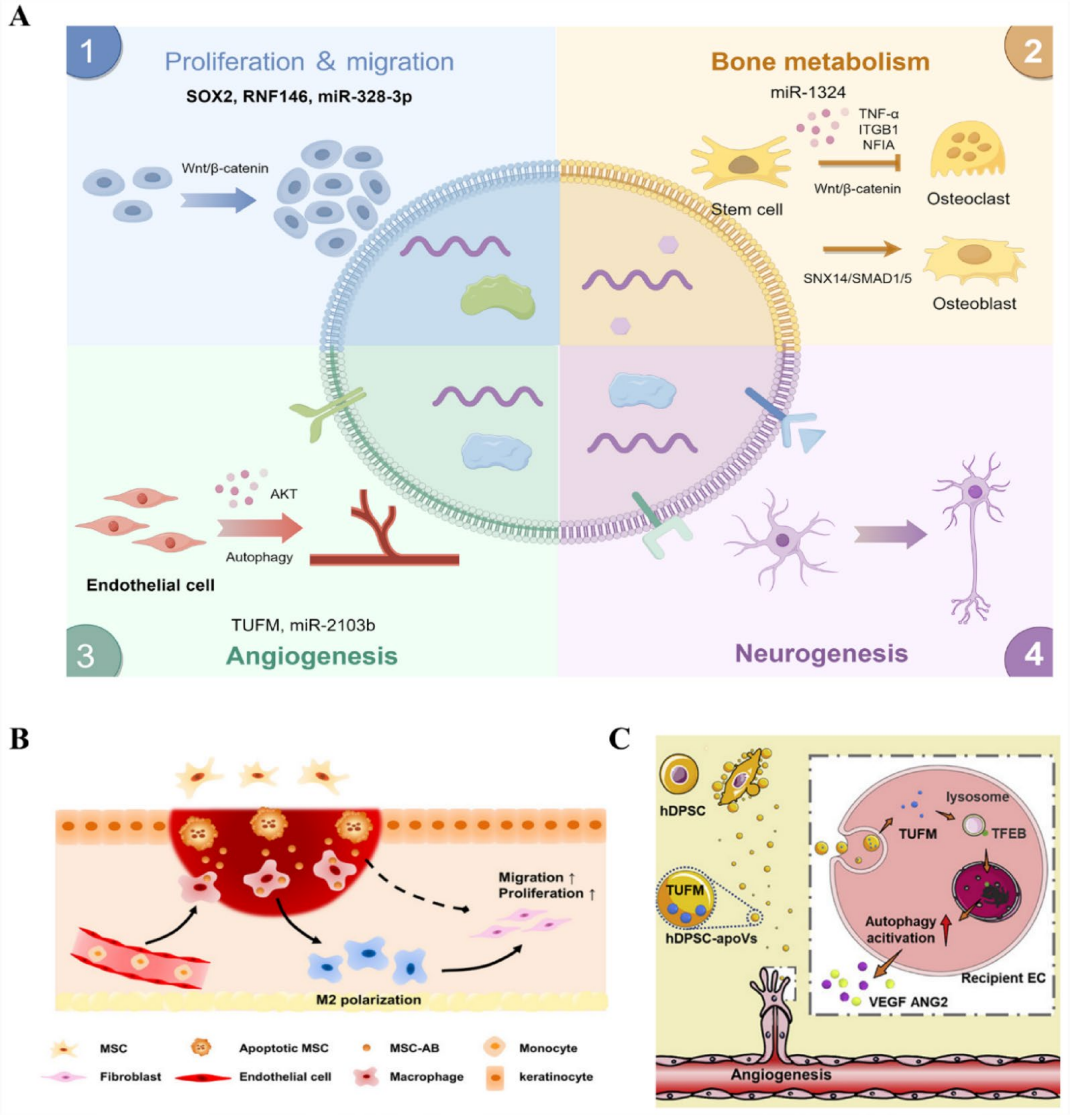
MSC-ApoVs have the potential to promote periodontal tissue regeneration. (**A**) Different components of MSC-ApoVs enhance precursor cell proliferation/migration, osteogenesis, angiogenesis, and neurogenesis but inhibit osteoclastogenesis. Created by Figdraw. (**B**) Schematic illustration of MSC-ApoVs converting macrophages towards the M2 phenotype and further enhancing the functions of fibroblasts. Reproduced with permission [130]. (**C**) Schematic illustration of MSC-ApoVs promoting endothelial cell (EC) autophagy by transferring TUFM to activate angiogenesis. Reproduced with permission [22]

The ultimate objective of periodontitis treatment is to achieve the repair and regeneration of periodontal tissues, focusing on forming functional soft and hard tissue composite structures. This includes promoting the regeneration and reconstruction of cementum, periodontal ligament, and alveolar bone. In this process, the biological activities of cell proliferation, migration, osteogenesis, angiogenesis, and related behaviors play crucial roles **(**Fig. 6A**)** [209]. The proliferation and migration of stem cells are critical for the repair and regeneration of periodontal tissues. Studies have shown that MSC-ApoVs can significantly enhance stem cell functions by activating specific signaling pathways. These vesicles contain signaling molecules that promote cell division, such as SOX2, RNF146, and miR-328-3p, which can activate the Wnt/β-catenin and Hippo signaling pathways upon ingestion by stem cells, thereby enhancing stem cell proliferation and migration capabilities to promote tissue regeneration [121, 210, 211]. Lai et al. discovered that MSC-ApoVs enhance the proliferation and migration of skin stem cells by promoting mitochondrial transfer, thereby ameliorating impaired cutaneous wound healing [144]. Additionally, the vivo preconditioning of MSC-ApoVs under hypoxic conditions enhances their miRNAs and active proteins, such as miR-1246, which further boost the proliferation and migration efficiency of stem cells through the activation of the Wnt and MAPK signaling pathways [147]. In future research on periodontal tissue regeneration, the activation of these signaling pathways can provide the driving force for stem cell proliferation and migration, effectively accelerating the natural repair process of damaged tissues **(**Fig. 6B**).** During periodontal tissue regeneration, the regulation of bone metabolism is crucial alongside stem cell proliferation and migration. Effective regulation of osteoclast generation and activity, and maintaining bone metabolic balance, are key to preventing alveolar bone loss [44, 212, 213]. Recent doubleblind, randomized, self-controlled clinical trials have shown that MSC-ApoVs accelerate alveolarbone regeneration, underscoring their considerable clinical potential for periodontal tissue repair [163]. Pre-clinical studies have shown that MSC-ApoVs can inhibit the shift of *Porphyromonas gingivalis*-LPS-induced macrophages to a pro-inflammatory phenotype and reduce TNF-α secretion by activating the AMPK/SIRT1/NF-κB signaling pathway, thereby inhibiting osteoclast formation [29]. Furthermore, Li et al. found that in a mouse periodontitis model, MSC-ApoVs are phagocytosed by osteoclasts via DC-STAMP and transfer miR-223-3p intracellularly, inhibiting the expression of NFIA and ITGB1, thereby interfering with osteoclast differentiation and function, reducing alveolar bone resorption [134]. Additionally, the enhancement of BMSCs’ function is also a key factor in regulating bone metabolism and supporting periodontal tissue regeneration [214–216]. For instance, Zhu et al. demonstrated that MSC-ApoVs can significantly enhance bone regeneration in vivo within animal bone defect models, improving both bone mass and bone density, and substantially boosting the osteogenic capabilities of native BMSCs [170]. In vitro studies revealed that MSC-ApoVs, by releasing miR-1324, inhibit the expression of the target gene sorting nexin 14 (SNX14), activate the SMAD1/5 signaling pathway, and bidirectionally regulate bone formation and resorption, thus effectively managing bone metabolism and promoting tissue regeneration. Liu et al. further showed that MSC-ApoVs released by BMSCs in areas of cranial defect significantly enhance the proliferation, migration, and osteogenic differentiation of endogenous BMSCs [121]. Research has also shown that MSC-ApoVs can regulate inflammation by suppressing the release of TNF-α in pro-inflammatory macrophages, which in turn inhibits the formation of osteoclasts, thus alleviating inflammation and alveolar bone loss during periodontitis. In the same animal model, Li et al. demonstrated that MSC-ApoVs promote new bone formation by increasing intracellular reactive oxygen species, which in turn activate the JNK signaling pathway [165]. In the osteoporotic mouse model, ApoVs activate the Ras/Raf1/Mek/Erk pathway via the Ras protein, promoting osteogenesis and bone formation both in vitro and in vivo [166]. Furthermore, some researchers have indicated that MSC-ApoVs facilitate osteogenesis by activating the extracellular signal-regulated kinase (ERK)1/2 signaling pathway [164]. Similarly, Zhang et al. showed that MSC-ApoVs containing miR-145a-5p significantly alleviate osteoporosis. These ApoVs enhance the osteogenic differentiation and immunomodulatory abilities of ovariectomy (OVX) MSCs by activating the TGF-β1/Smad 2/3 and Wnt/β-catenin signaling pathways compared to OVX MSC-ApoVs lacking miR-145a-5p [124]. Importantly, bone immunity is critical in the bone metabolic process during the regeneration of periodontal tissues, especially by promoting the suppression of osteoclast formation by reducing M1 polarization and fostering bone regeneration through the polarization of M2 macrophages. Previous discussions have indicated that MSC-ApoVs can exert a positive effect on this bone immune process through their immunoregulatory properties. Overall, MSC-ApoVs enhance bone tissue regeneration and repair not only through direct modulation of cell functions but also via immunomodulatory effects, underscoring their potential utility in periodontal hard tissue regeneration. Additionally, MSC-ApoVs enhance the function of fibroblasts to promote soft tissue regeneration. In a study by Liu et al., mice with skin injuries injected with BMSC-ApoVs showed faster wound healing and more complete skin structure reconstruction. This repair effect is likely due to the indirect effects of BMSC-ApoVs on fibroblasts, particularly their ability to promote the polarization of macrophages towards an M2 type, which in turn enhances fibroblast proliferation and migration, accelerating skin wound healing [130]. Given that fibroblasts are a key cellular component of periodontal soft tissues, these findings underscore the potential application of MSC-ApoVs in promoting the regeneration of periodontal soft tissues. In the regeneration of periodontal tissues, angiogenesis is crucial, ensuring adequate blood supply for the transfer of oxygen and nutrients, with MSC-ApoVs playing a key role in this process [217]. Studies indicate that after uptake by endothelial cells (ECs), MSC-ApoVs can enhance the expression of genes related to angiogenesis through autophagy mechanisms, thereby promoting neovascularization and supporting the reconstruction of dental pulp blood supply and tissue regeneration (Fig. 6C) [22]. Moreover, in myocardial infarction models, MSC-ApoVs activate ECs’ lysosomes, increase lysosome-associated membrane protein 1 expression, and facilitate the nuclear translocation of transcription factor EB, enhancing angiogenic capabilities while also promoting autophagy in damaged myocardial cells to improve heart function  [218]. Further, MSC-ApoVs enriched with miR-210-3p promote EC migration by upregulating AKT expression, effectively enhancing angiogenesis and aiding wound healing [148].

Furthermore, research indicates that MSC-ApoVs promote vascular remodeling and inhibit pathological angiogenesis by modulating endothelial cell glycolysis through the PD1/PDL1 axis, thereby rescuing ischemic retinal diseases [168]. Similarly, Fei’s research demonstrated that MSC-ApoVs facilitate angiogenesis by transferring the functional molecule COL1A1 and activating the PI3K/Akt/VEGF pathway [169]. In addition to directly promoting angiogenesis, MSC-ApoVs can modulate endothelial cell function, enhancing the recruitment of endogenous somatic cells and facilitating tissue regeneration and repair. Lu’s study found that MSC-ApoVs enriched with oxidized phosphatidylcholine activate endoplasmic reticulum stress in endothelial cells, which leads to the increased expression of adhesion molecules. This process induces the recruitment of autologous stem cells within blood vessels, promoting osteogenesis and repair of femoral defects in mice, thus revealing the mechanism by which allogeneic stem cells promote bone repair [167]. These studies highlight the potential role of MSC-ApoVs in regulating EC functions and promoting angiogenesis, significantly enhancing the possibilities for periodontal tissue regeneration.

Neurogenesis is a key strategy for achieving effective tissue regeneration in periodontal therapy. Nerve fibers can promote angiogenesis, modulate immune responses, and directly influence fibroblasts and osteoblasts by releasing neuropeptides and growth factors, thus contributing to the regeneration and healing of periodontal tissues [219–221]. Recent studies are exploring the use of EVs and growth factors to enhance bone and periodontal tissue regeneration through neurogenesis [217, 222, 223]. Research indicates that MSC-ApoVs can treat nerve damage caused by cerebral ischemia-reperfusion by scavenging excess ROS, reducing oxidative stress and apoptosis, and enhancing neural cell activity. Additionally, proteomic analysis shows that MSC-ApoVs contain functional proteins related to neuroprotection, demonstrating their potential applications in neurogenesis [171]. Since neurovascular coupling is crucial for bone tissue repair and regeneration, and neurovascularized bone regeneration is vital for alveolar tissue regeneration, further strengthening research in this area will facilitate neurovascularized periodontal tissue regeneration [224, 225].

Therefore, synthesizing the current research data, we can reasonably speculate that MSC-ApoVs show robust potential in promoting the proliferation and migration of stem cells in periodontal tissues, regulating bone metabolism, as well as enhancing angiogenesis and neurogenesis. These actions collectively contribute to the effective repair and regeneration of periodontal tissues.

### MSC-ApoVs for drug delivery in periodontal tissues

**Fig. 7:**
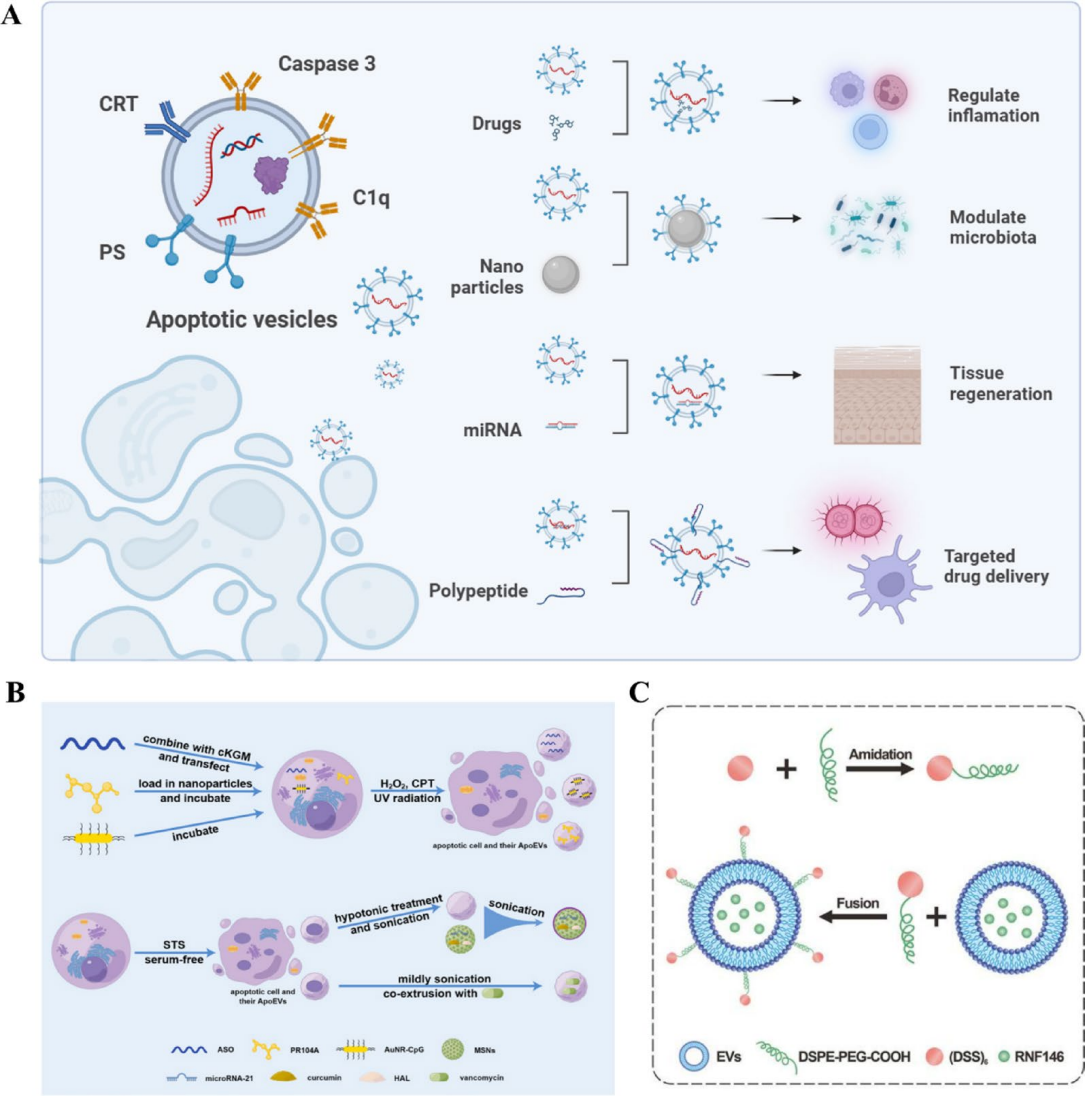
MSC-ApoVs as an effective drug delivery system. (**A**) MSC-ApoVs, after bioengineering modifications, can significantly improve their targeting accuracy and therapeutic effects by loading specific drugs, nanoparticles, or mRNA, or through surface modifications. Created with BioRender.com. (**B**) Cells can be loaded with cargo either before or after inducing apoptosis to produce engineered ApoVs. Reproduced with permission [93]. (**C**) Schematic diagram of the protocol for producing the ApoVs delivery system. Reproduced with permission [172]

Recently, EVs’-based drug delivery systems for the treatment of periodontitis have seen rapid development. This precise method of administration helps reduce issues of drug resistance, prevents complications associated with broad-spectrum treatments, and enhances therapeutic efficacy and outcomes [226, 227]. As a type of extracellular vesicle extensively studied recently, modified MSC-ApoVs not only achieve more precise immunoregulation but also aid in tissue regeneration and exhibit effective antimicrobial functions, all crucial for periodontitis treatment **(**Fig. 7A**)** [28]. MSC-ApoVs can encapsulate specific therapeutic molecules, such as microRNA and drugs, in various ways to effectively target specific therapeutic areas, optimizing treatment outcomes and reducing side effects [93] **(**Fig. 7B**)**. Research by Geng et al. showed that nanoparticles loaded with microRNA-21 and curcumin embedded into ApoVs effectively targeted macrophages to promote their M2 polarization, modulated inflammatory responses, reduced the expression of inflammatory factors, and aided in symptom improvement in inflammatory bowel disease [173]. Another study combined anti-TNF-α antisense oligonucleotides with cationic konjac glucomannan and utilized transfection/apoptosis induction processes to load these into ApoVs, achieving drug delivery through the blood-brain barrier to suppress brain inflammation and treat Parkinson’s disease [228]. Xin used electroporation to load triptolide into ApoVs, increasing the targeted delivery efficiency and biocompatibility of the drug, which in turn modulated macrophage autophagy and polarization, effectively treating age-related xerostomia [229]. Gui et al. used a pre-secretion strategy to integrate E3 ubiquitin ligase RNF146 into natural MSC-ApoVs, activating the Wnt/β-catenin pathway and significantly enhancing osteogenic efficacy **(**Fig. 7C) [172]. These studies indicate that MSC-ApoVs substantially improve treatment outcomes for diseases through their precise drug delivery capabilities. Previous studies have also revealed an efficient drug-loading strategy where apoptosis induced by specific drugs such as α-Mangostin (α-M) can directly integrate therapeutic agents into ApoVs. It not only effectively regulates immune responses but also enhances angiogenesis and cell proliferation, playing a significant role in anti-inflammation and tissue regeneration [171]. This strategy provides a novel pathway for using ApoVs to regulate inflammation and repair tissues, demonstrating potential clinical applications. Notably, in addition to adding elements, subtracting components from ApoVs can also enhance therapeutic effects. Cheng’s study demonstrated that downregulating hsa-miR-4485-3p in MSC-ApoVs, akin to releasing a brake, resulted in more potent osteoinductive ApoVs. This enhancement is likely related to hsa-miR-4485-3p’s ability to inhibit osteogenesis by targeting the AKT pathway [230].

Nanoparticle technology is recognized as an efficient loading method in the field of drug delivery [231, 232]. During apoptosis, nanoparticles such as polycarbonate (PC), liposomes, and mesoporous silica nanoparticles can be introduced to form an efficient encapsulated system of ApoVs-nanoparticles. Research shows that the synthesis of ApoV-nanoparticle systems is more efficient during mid-apoptosis. By integrating Bortezomib (BTZ) with polycarbonate into proliferating apoptotic MSCs, the resulting BTZ/PC-ApoVs system not only exhibits a synergistic effect between BTZ and ApoVs but also delivers better therapeutic results in a mouse model of multiple myeloma. This system significantly decreases bone damage and increases bone mass and the bone volume/total volume, thus improving the disease’s pathological status and markedly lowering BTZ’s systemic toxicity. Furthermore, Rab7 plays a crucial role in regulating the efficiency of nanoparticle encapsulation within apoptotic MSCs; its activation enhances the production of nanoparticle-ApoVs, providing a significant biological basis for improving encapsulation efficiency [233]. Besides loading nanoparticles inside ApoVs, researchers have discovered that nanoparticles can bind to MSC-ApoVs through electrostatic forces. Specifically, negatively charged copper-doped carbon dots (Cu-CDs) with excellent antibacterial properties can combine with ApoVs, which possess targeting advantages, to form CC-ApoVs. This combination enhances the therapeutic effect beyond that of Cu-CDs or ApoVs alone, effectively rescuing damaged Kupffer cells during sepsis [175]. These studies highlight the potential of the ApoV-nanoparticle system in drug delivery, particularly in regulating local inflammatory responses and promoting tissue regeneration. This technology enables more effective direct drug delivery to inflamed or diseased areas, reducing systemic side effects and enhancing therapeutic efficiency, offering a new direction for future disease treatment strategies.

Through a specialized targeting mechanism, modified ApoVs can specifically kill pathogenic microorganisms, demonstrating potential as “nano-decoys”. In experiments, vancomycin-loaded ApoVs not only kill macrophages and cancer cells infected with *Staphylococcus aureus* but also deliver vancomycin inside the cells by targeting the “eat me” signal. This significantly enhances the efficiency of drug delivery and reduces colony-forming units, proving their advantage in treating *Staphylococcus aureus* infections [174]. To date, the periodontitis field lacks head-to-head comparisons between vesicle-encapsulated antibiotics and standard local antimicrobial therapies. Common oral antiseptics, such as chlorhexidine, maintain contact with bacteria for only 60–90 s; chlorhexidine and metronidazole placed in periodontal pockets are typically washed out within 24–48 h and must be reapplied daily [234, 235]. By contrast, vancomycin-loaded ApoVs released drug continuously for 72 h in an invitro S. aureus–macrophage model, reducing colony-forming units (CFUs) by > 2 log, whereas an equal dose of free vancomycin cut CFUs by < 1 log [174]. These findings indicate that the ApoV platform prolongs antimicrobial activity and enhances intracellular killing capacity, yet rigorous animal studies are still needed for verification. Surface modification of ApoVs can enhance their targeting and drug-loading efficiency. For example, You et al. introduced a matrix metalloproteinase-activated cell-penetrating peptide onto the surface of MSC-ApoVs, enhancing their targeting under ischemic brain conditions. This design allows ApoVs’ uptake only when MMP9 is activated, enabling precise treatment and reducing the risks associated with systemic administration [171]. Additionally, researchers coupled a bone-targeting peptide (Asp-Ser-Ser)_6_ to MSC-ApoVs using DSPE-PEG-COOH as a linker, enhancing their bone tissue targeting. These engineered ApoVs maintain the biocompatibility and natural properties of ApoVs while significantly boosting their osteogenic effects, particularly showing excellent results in the treatment of osteoporosis [172]. Although engineered MSC-derived ApoVs hold therapeutic promise, they may be taken up by off-target tissues and trigger immune activation. Even with MSCs’ inherently low immunogenicity, MSC-ApoVs from allo or xenogenic sources may still be perceived as foreign, provoking complement cascades, proinflammatory cytokine bursts, or anti-vesicle antibody formation [236]. To mitigate these risks, “don’t eat me” ligands can be grafted onto ApoVs’ surfaces to diminish macrophage uptake and increase targettissue accumulation, while gene editing can reduce MSC MHCI expression to further lower immunogenicity [237, 238]. Additionally, tethering ApoVs to scaffolds or embedding them in slow-release matrices can localize their action to periodontal lesions and minimize systemic spread [239]. Their safety must be thoroughly and repeatedly validated prior to clinical deployment.

**Fig. 8:**
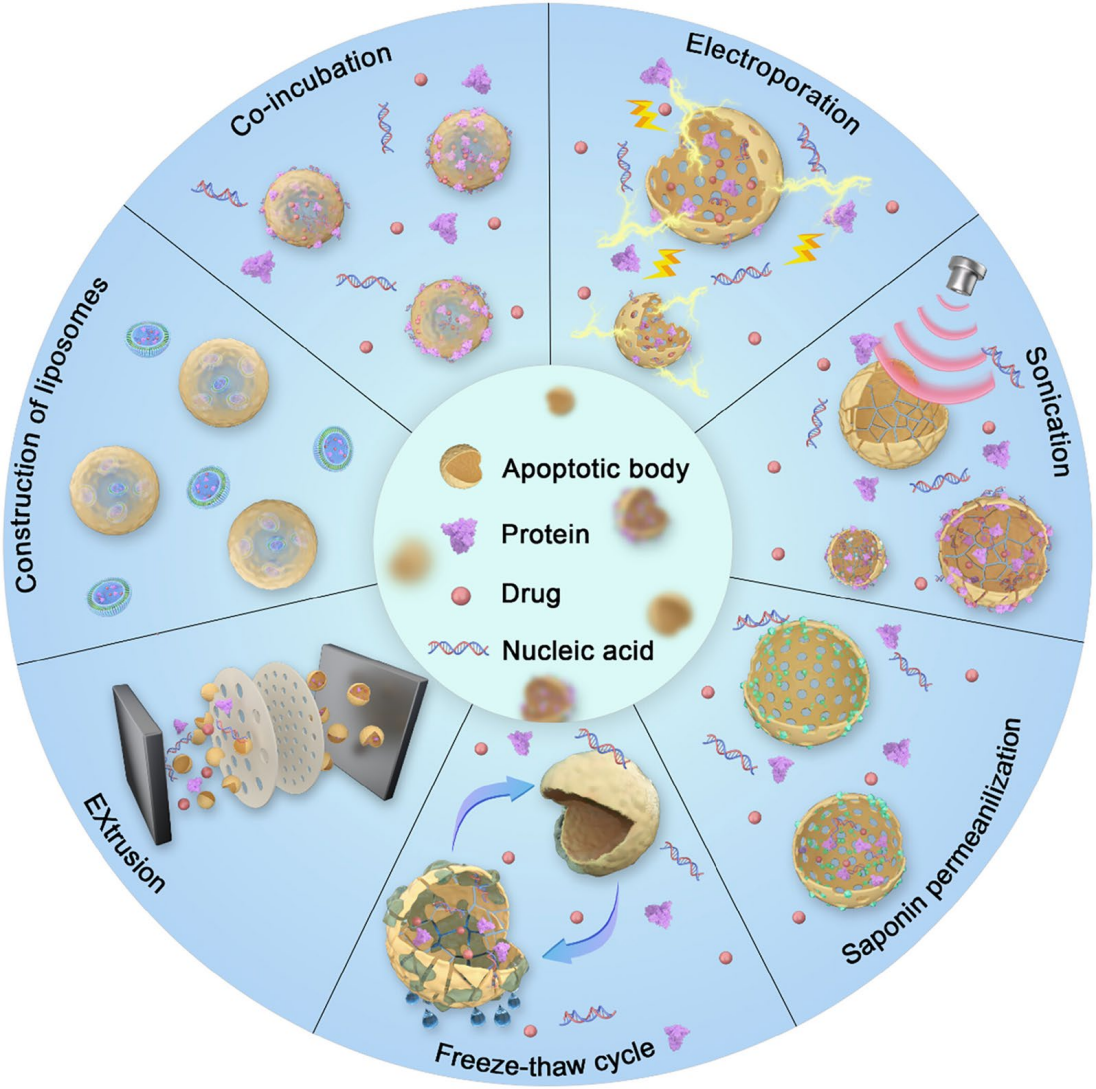
Technical methods for loading drugs into ApoVs. Reproduced with permission [138]

Multiple modification techniques for ApoVs are now available (Fig. 8**)** [138]. Although ApoVs have been extensively studied and proven effective as drug delivery platforms, especially due to their unique surface markers that promote phagocytosis by phagocytic cells, they show great potential to become superior biopharmaceutical delivery platforms. However, the research on drug delivery using MSC-ApoVs is still in its early stages and requires further exploration. The ease of in vitro cultivation of MSCs and the high yield and excellent safety of MSC-ApoVs indicate their pronounced prospect for drug loading, especially suitable for carrying anti-inflammatory drugs, antimicrobial agents, or specific siRNAs, offering new strategies for periodontal disease treatment.

### MSC-ApoVs-based strategies for periodontitis treatment

**Fig. 9:**
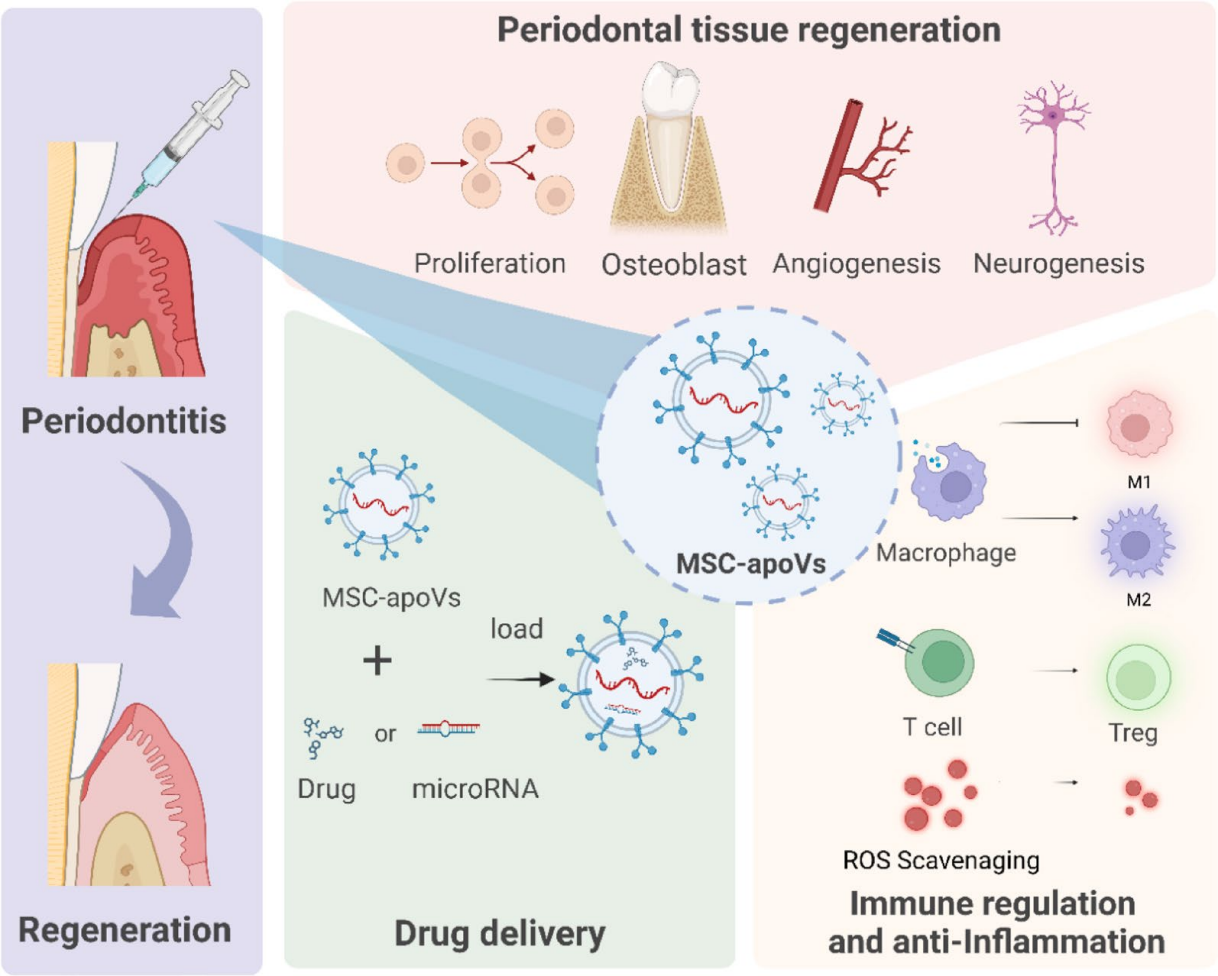
Potential mechanisms of MSC-ApoVs-based periodontitis treatment. Created with BioRender.com

Based on prior research, MSC-derived ApoVs hold significant promise in periodontitis therapy through immunomodulation, enhancement of tissue regeneration, and targeted drug delivery. Immunologically, MSC-ApoVs encourage polarization towards M2 macrophages, decrease the tendency towards M1 macrophages, and lower the expression of related inflammatory markers. Moreover, they might enhance the microenvironment of neutrophils through certain mechanisms, thereby curtailing the release of inflammatory factors or mitigating ROS release to diminish oxidative stress in the tissues. Additionally, MSC-ApoVs can carry functional proteins or mRNA from their parent cells, which facilitates cell proliferation and migration via the Wnt/β-catenin pathway. They also support the osteogenic differentiation of various stem cells, the angiogenic differentiation of endothelial cells, and neurogenesis, thereby enabling neurovascularized periodontal tissue regeneration (Fig. 9). MSC-ApoVs exhibit outstanding stability due to their unique lipid bilayer encapsulation, maintaining consistent particle size even after treatment with phosphate-buffered saline and fetal bovine serum [228].

Contemporary pharmacotherapy for periodontitis predominantly employs local antimicrobials, systemic antibiotics, and anti-inflammatory drugs. However, their therapeutic performance, stability, and target selectivity are inadequate for patient needs, and they incur certain sideeffects [240]. Appropriately engineered ApoVs carrying specific miRNAs or bioactive compounds can finely tune immune and bacterial functions, suppressing host inflammation and plaque development, or alternatively boost PDLSC regenerative capacity, thus potentiating ApoV-based therapy and bolstering periodontal treatment outcomes. Relative to exosomes, ApoVs act as a more competitive vesicle platform, offering superior loading efficiency and wider drug versatility, which supports these advanced therapeutic applications [233]. In contrast to exosomes, ApoVs possess a diverse size range and an expanded functional repertoire. Miniature ApoVs lend themselves to precise payload delivery, while larger counterparts are more readily phagocytosed and may serve as phagocyte-directed vectors [110]. Meticulous structural engineering of MSC-ApoVs could therefore amplify both their efficacy and specificity. Prospectively, surface engineering of ApoVs with ligands targeting periodontal pathogens or specific resident cells promises to refine their accuracy in periodontitis treatment.

**Fig. 10:**
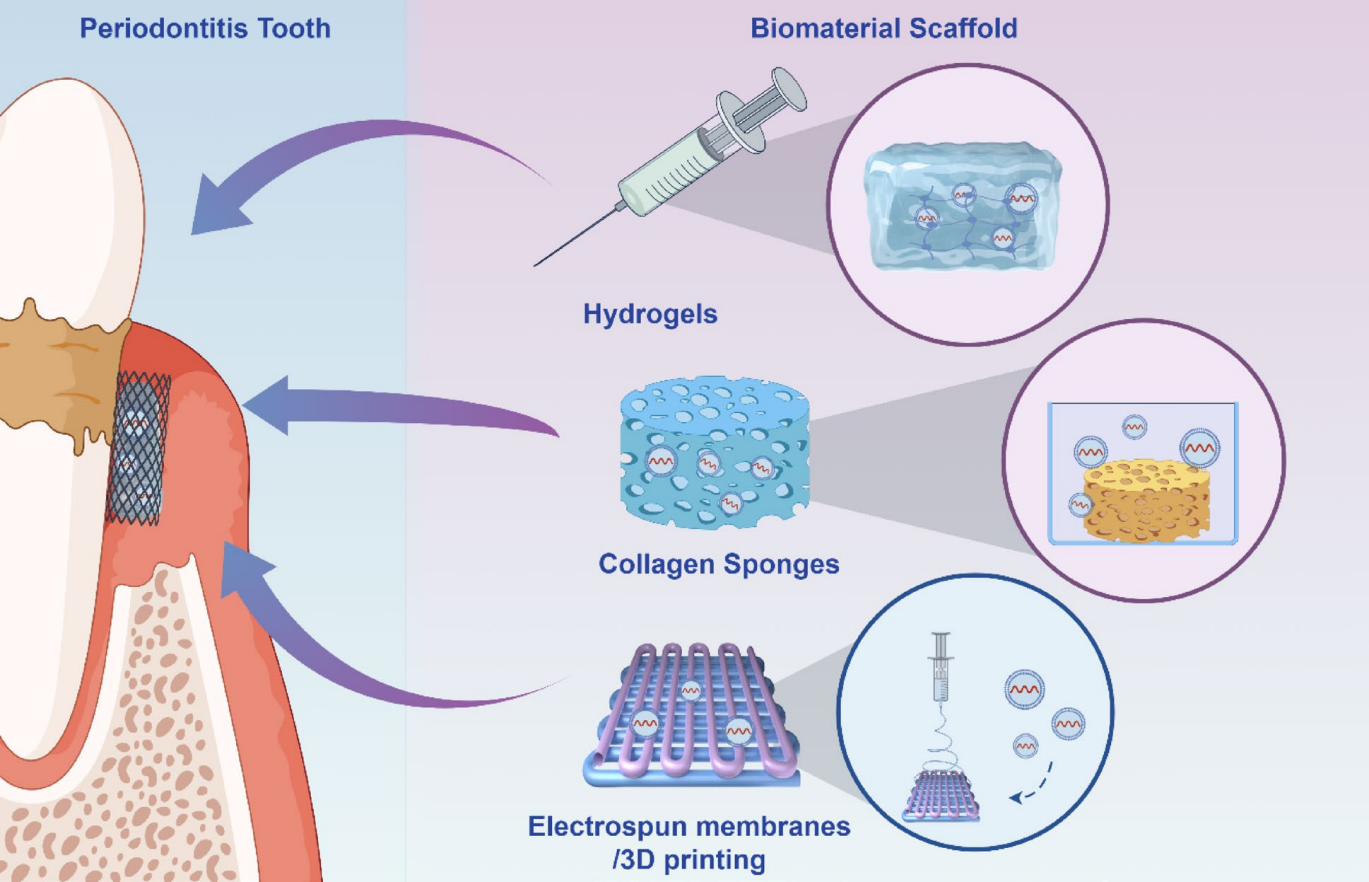
MSC-ApoVs can be combined with a variety of biomaterial scaffolds to enhance their stability and slow-release properties for the treatment of periodontitis and periodontal defects. Created by Figdraw

Current studies highlight local delivery as the principal strategy for targeting periodontal tissues, with biomaterials providing additional retention and therapeutic benefits. Evidence indicates that locally injected MSC-ApoVs bearing a DCSTAMP ligand, which binds selectively to receptors on alveolar pre-osteoclasts and triggers their internalization, thereby directly modulating alveolar bone resorption [134]. In addition, bonetargeting peptide modification, via conjugation of the (Asp-Ser-Ser)_6_ sequence to the vesicle surface, further enhances ApoVs accumulation within bone by exploiting hydroxyapatite affinity [172]. Moreover, when vesicles are simply instilled, salivary flow and mechanical forces cause rapid wash-out, precluding sustained therapy; by contrast, biomaterials such as injectable hyaluronic-acid hydrogels, lyophilized adhesive sponges and electrospun nanofiber membranes can efficiently encapsulate ApoVs, prolong their retention in periodontal pockets, markedly reduce systemic dissemination and preserve vesicle integrity [241, 242], thereby maximizing on-target action and minimizing off-target effects. Furthermore, implanting membrane vesicles alone into diseased periodontal sites provides limited regeneration, because newly formed cells lack adequate space and a supportive micro-environment [227]. Biomaterial scaffolds can guide organized tissue regeneration and furnish a physiological periodontal niche that supports cell proliferation [243, 244]. Importantly, ApoVs offer distinctive benefits; laden with pro-coagulant PS and tissue factor, MSC-ApoVs integrated with scaffold materials have been shown to arrest bleeding swiftly, a clinically meaningful feature for periodontitis patients troubled by gingival hemorrhage or post-surgical bleeding [245]( Fig.10).

**Fig. 11:**
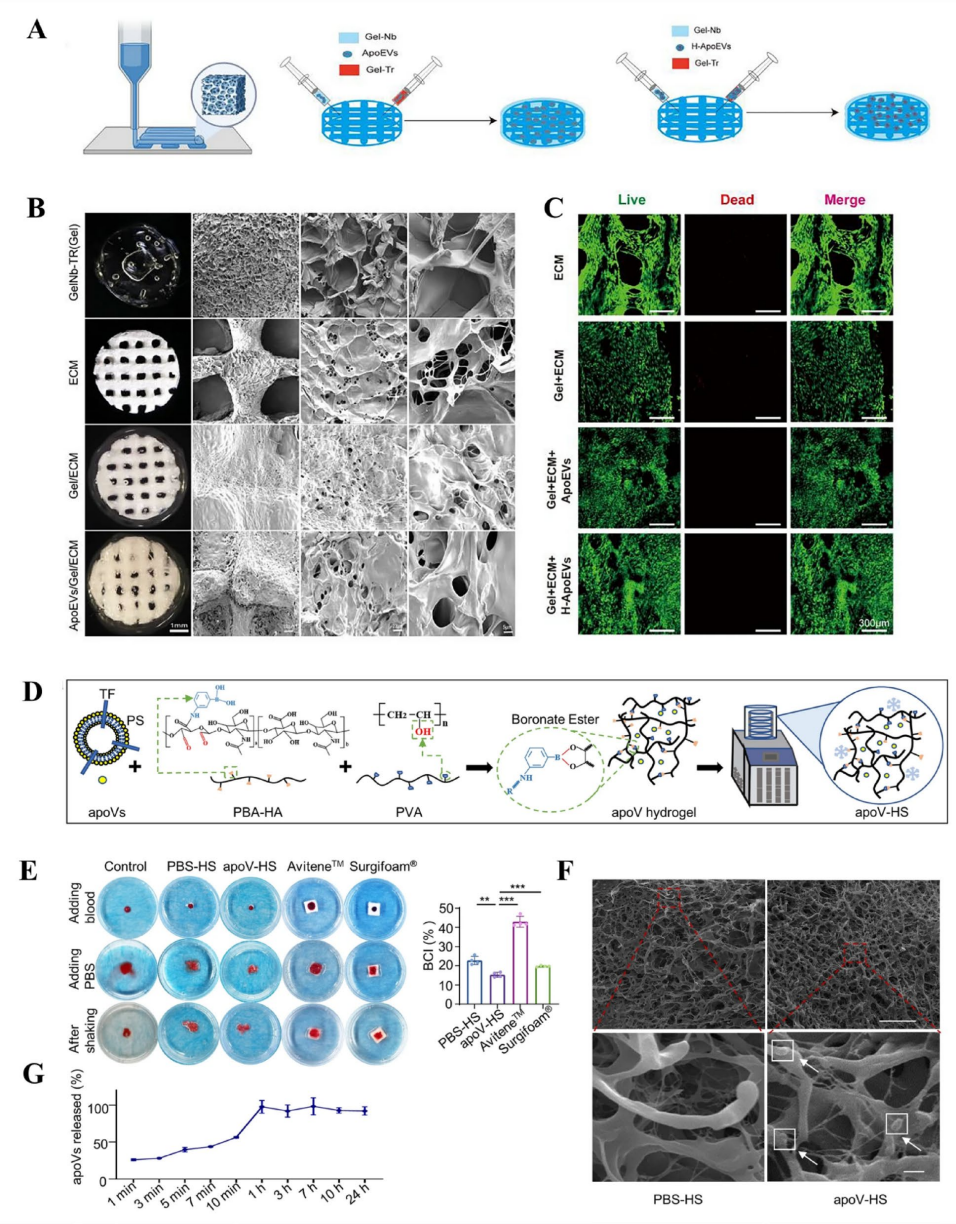
Loading strategies of ApoVs with different biomaterials. (A) Schematic illustration of the preparation of a Gel/ECM composite scaffold loaded with ApoVs. (B) Macroscopic and SEM images of GelNb-TR, ECM, Gel/ECM, and Gel/ECM composite scaffolds loaded with ApoVs. (C) Fluorescence staining to analyze BMSC viability after 7 days of culture on ECM, Gel/ECM, Gel/ECM composite scaffolds loaded with ApoVs. Reproduced with permission [147]. (D) Schematic diagram indicating the assembly of ApoVs-encapsulated hydrogel sponge (ApoV-HS) and the procedures of lyophilization. (E) Digital photos illustrating the process of blood clotting upon hemostatic agents and quantification of the blood clotting index (BCI). (F) Scanning electron microscopy (SEM) images showing the morphology of the lyophilized hydrogel sponge after hydration. (G) Quantification of ApoVs’ release efficiency from ApoV-HS. n = 4 per group. Reproduced with permission [245]

Hydrogels, due to their excellent biocompatibility and tunability, are commonly used for minimally invasive drug delivery and are important materials in tissue engineering. In regenerative medicine, hydrogels are used as scaffolds, barriers, drug delivery platforms, and cell encapsulation matrices [246, 247]. Various biopolymer hydrogels loaded with ApoVs have been applied in vivo studies. For example, some studies have used modified gelatin and 3D-printed extracellular matrix (ECM) scaffolds to deliver MSC-ApoVs. This Gel/ECM composite scaffold, with its excellent biocompatibility and ability to fill tissue defects, can sustainably release ApoVs for over a week (Fig. 11A-C**)** [147]. Another study developed a local delivery system of hydrogel microspheres containing MSC-ApoVs. These microspheres, prepared by a coaxial microfluidic device, preloaded GelMA-alginate microspheres (GA-MSP@MSC-ApoVs) that can precisely control the release of MSC-ApoVs, aligning with the critical periods of tissue healing and neovascularization to optimize therapeutic effects [148]. It is worth emphasizing that researchers have developed an ApoV hydrogel with high productivity, physiological stability, injectability, plasticity, excellent adhesivity, biocompatibility, and rapid coagulation properties. Specifically, they combined ApoVs with phenylboronic acid-grafted oxidized hyaluronic acid and polyvinyl alcohol, then used lyophilization to construct a novel ApoV-encapsulated hydrogel sponge (ApoV-HS). This innovative material is well-suited for the clinical hemostatic treatment of periodontitis (Fig. 11D-F**)** [245]. These innovative strategies have enhanced the stability and bioavailability of active factors during the therapeutic process, providing significant technical support for the clinical application of MSC-ApoVs in periodontitis.

Collagen sponges, as natural extracellular matrix-derived materials, are widely used in periodontal regeneration due to their excellent biocompatibility, absorbability, and inertness, making them ideal biological scaffolds for extracellular vesicles [248]. Their rapid absorbability helps prevent periodontal defect infections and promotes tissue regeneration [249]. For example, researchers cut collagen sponges into 3 × 2 × 1 mm blocks and loaded them with extracellular vesicles. The results showed that the collagen sponges had an orderly framework and regular pore distribution, with vesicles of approximately 100 nm in diameter attaching to the material. Animal experiments have demonstrated that collagen sponges loaded with outer membrane vesicles have a significant effect on periodontitis, promoting alveolar bone regeneration [239].

The biomembrane plays a crucial role in periodontal tissue regeneration, particularly in guided tissue regeneration (GTR) [250–252]. After conventional periodontal disease treatment, the inner wall of the periodontal pocket and the exposed root surface often heal by forming long epithelial attachments, but they lack functional periodontal ligament fibers. True periodontal regeneration requires the formation of a new periodontal ligament, cementum, and alveolar bone, with periodontal ligament fibers connecting to the newly formed alveolar bone [253]. The GTR barrier membrane prevents the growth of gingival epithelium and connective tissue along the root surface, providing space to guide periodontal membrane cells to preferentially occupy the root surface. This promotes the formation of cementum and periodontal membrane fibers, achieving periodontal tissue regeneration. Nanofiber membranes prepared through electrospinning technology are widely used in GTR [252, 254]. These membranes mimic the structure and morphology of ECM proteins, promoting cell adhesion, proliferation, and differentiation [255–259].

Studies have shown that these membranes, when loaded with extracellular vesicles, can effectively adsorb the vesicles due to their high porosity, thereby slowing the release of vesicles and enabling disease treatment [260]. Researchers combined MSC-EVs with phosphoethanolamine phospholipid-grafted poly-L-lactic acid micro/nanofibers (DSPE-PLLA). This combination significantly slowed down the local vesicle release, promoting tissue repair and improving wound healing by alleviating inflammation, and stimulating cell proliferation, collagen deposition, and angiogenesis [261]. Similarly, Li et al. applied a layer of functionalized polydopamine on electrospun nanofibers, enhancing the incorporation of MSC-EVs through synergistic interactions of adhesion force, hydrogen bonding, and electrostatic interaction, allowing for localized sustained delivery of outer membrane vesicles to achieve therapeutic effects.

In addition, with the advancement of technology, 3D printing has been extensively studied in periodontal tissue regeneration. This approach enables the construction of highly precise biological scaffolds that effectively mimic natural periodontal structures and support the regeneration of bones and periodontal ligaments. By utilizing a variety of biomaterials and EVs, 3D printing can provide personalized regeneration solutions, significantly enhancing therapeutic outcomes. For example, Sun et al. demonstrated that a porous scaffold constructed using 3D printing with β-tricalcium phosphate (β-TCP) bioceramics and EVs can achieve predefined structures and sustained release of exosomes [262]. Similarly, researchers have incorporated MSC-derived EVs into a mixture of hydrogels and decellularized ECM for 3D printing. This creates osteochondral scaffolds with spatially biomimetic structures that promote attachment, spreading, migration, proliferation, chondrogenesis, and osteogenic differentiation of rat BMSCs in vitro. As a result, these scaffolds facilitate cartilage and bone repair in osteochondral defects [263].

Collagen sponges and barrier membranes, including products such as Geistlich BioGide^®^, BioOss^®^ Collagen, Zimmer CopiOs^®^, and Helistat^®^, have already seen broad clinical use as adjuncts for guided tissue regeneration in treating intrabony periodontal defects [264, 265]. However, integrating MSC-ApoVs with such scaffolds is still confined mainly to preclinical research. In a rat periodontitis model, collagen sponges loaded with membrane vesicles significantly increased alveolarbone height and density [239]. Shi et al. embedded LPS-primed dentalfollicle exosomes in electrospun GTR nanofibres and achieved superior periodontal regeneration in rodent models [78], yet ApoVs have not been evaluated in similar studies. An injectable gelatin–ECM hydrogel encapsulating MSC-ApoVs boosted stemcell proliferation and osteogenesis in a calvarialdefect model, but has yet to be tested in periodontitis [147]. Scaffold–ApoVs composites show promise in pre-clinical bone and tissuerepair studies, yet their safety, local retention, and regenerative efficacy must be tested in dedicated periodontitis models to advance clinical translation.

Although the study of combining ApoVs and scaffolds is still in its early stages, ample evidence from existing EVs research shows that this combination can significantly enhance the stability and sustained release properties of the vesicles, thus increasing their therapeutic potential. Moreover, it allows for the customization of scaffold platforms tailored to the periodontal defects of periodontitis patients, thereby enhancing their therapeutic potential [266–269]. This enhanced stability and prolonged release period provide significant advantages for the clinical application of ApoVs, especially in achieving sustained therapy and promoting periodontal tissue repair. Therefore, further research and development of this technology are expected to promote ApoVs as an effective approach for periodontal regeneration therapy. The application of ApoVs can be customized based on the functional characteristics of the MSCs they originate from and the specific pathological condition of the patient, thus achieving precise treatment. For example, during the acute inflammatory stage of periodontitis, patients can use ApoVs with significant anti-inflammatory functions to modulate macrophage polarization and effectively reduce the inflammatory response. In contrast, for patients with chronic periodontitis and severe bone tissue defects, the main treatment goal is to repair the damaged alveolar bone. In this case, ApoVs with significant osteogenic activity can be selected. As genomics and bioinformatics progress, future periodontitis treatments may transition from traditional symptom-based classification to personalized treatment strategies based on genetic profiling. In this context, the selection of ApoVs will be determined by their protein and RNA contents, which can regulate the expression of specific genes. This approach can enhance therapeutic efficacy and achieve optimal personalized treatment by selecting bioactive materials tailored to the patient’s specific periodontal defect areas. Consequently, the application potential of ApoVs technology will be fully realized, further advancing its clinical application and development process.

**Fig. 12:**
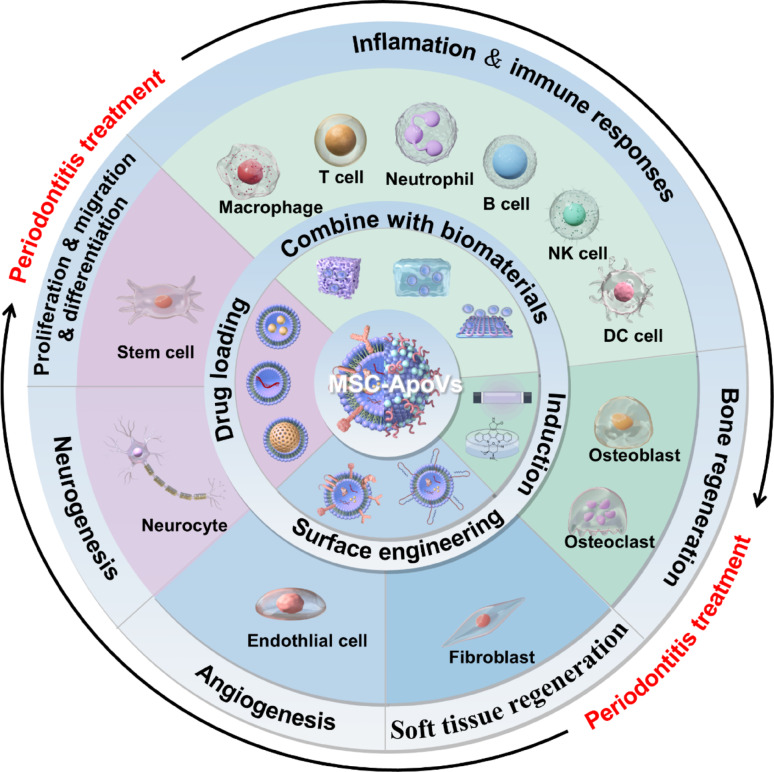
Illustration depicts MSC-ApoVs tailoring and effects of MSC-ApoVs on functions of various cells present in periodontal tissues, which indicate tremendous application potential of MSC-ApoVs in periodontitis treatment.Created by Figdraw.

In conclusion, MSC-ApoVs have great clinical application potential in the treatment of periodontitis. By combining ApoVs with tissue engineering technology and loading them with specific drugs or nanoparticles, the targeting and specificity of ApoVs can be enhanced. MSC-ApoVs can be tailored to obtain the optimal therapeutic effects on different cells regulating inflammation, immune responses, cell functions (proliferation, migration, and differentiation), and multiple types of tissue regeneration, such as gingiva, periodontal ligaments, alveolar bone, blood vessels, and nerves (Fig. 12).

## Challenges and prospects

In recent years, the application of MSCs for the treatment of periodontitis has received widespread attention. Although MSC transplantation has shown potential in regenerating periodontal tissue, the inevitable issue of cell apoptosis in the short term remains a challenge. Consequently, researchers have delved into the mechanisms of MSC therapy and are increasingly focusing on using MSC-ApoVs as a novel treatment strategy. This approach aims to achieve optimal therapeutic effects for periodontitis by utilizing vesicles produced by apoptotic MSCs. MSC-ApoVs show significant advantages in treating periodontitis compared to direct cell transplantation. Firstly, their low immunogenicity reduces the risk of immune rejection [82, 270]. Secondly, as EVs, ApoVs are more convenient to store and transport than live cells [271]. Additionally, MSC-ApoVs serve as an effective drug delivery platform, supporting personalized treatment strategies while avoiding issues related to cell transplantation, such as cell apoptosis and ethical concerns [272, 273]. Notably, MSC-ApoVs also demonstrate great potential in drug delivery and vaccine development [28], making them strong candidates for developing cell-free therapeutic strategies and offering new possibilities for future treatments.

Pre-clinical and early clinical evidence indicates that ApoVs hold considerable translational promise for periodontitis therapy, yet no MSCApoV clinical trial targeting this disease has been launched to date. In a doubleblind, randomized, self-controlled trial, Jiang et al. reported that GMPgrade MSC-ApoVs markedly accelerated alveolarbone regeneration, while routine haematology and hepatic–renal panels showed no adverse reactions, underscoring their high biosafety profile [163]. In addition, a study of nonsurgical EV therapy for stage I–III periodontitis found significant post-treatment improvements in plaque index, bleeding on probing, probing depth, and clinical attachment level, accompanied by marked reductions in proinflammatory cytokines, further supporting the therapeutic potential of ApoVs [274]. Clinical studies of ADSCEVs for periodontitis (NCT04270006) and autologous DPSCEV transplantation for chronic periodontitis (ChiCTR1900027140) are presently ongoing and pending results. A separate phase I/II trial of grape-derived exosomes for preventing chemo/radiotherapy-induced oral mucositis (NCT01668849) has already demonstrated good tolerability and preliminary efficacy of EV therapy in oral diseases. Collectively, these pilot investigations not only verify the safety of ApoVs but also establish a solid foundation for future clinical trial design and broader deployment of MSC-ApoVs in periodontitis patients. MSC-ApoVs are easier to prepare, store, and scale up, and yield higher output [86]. Lyophilised MSC-ApoVs retained their bioactivity after three months at − 80 °C, 4 °C, or 25 °C, matching freshly isolated vesicles and indicating excellent storage stability and clinical promise [163]. Inducing apoptosis boosts vesicle yield from MSCs by roughly threefold, greatly enhancing production efficiency, whereas exosome isolation is more cumbersome and time-consuming [27]. By refining apoptosisinduction parameters and implementing standard operating procedures, MSC-ApoVs production can be scaled up while limiting batch variability, thereby furnishing a solid basis for industrial-level manufacture and consistent quality.

Despite the promising clinical outlook of MSC-ApoVs, challenges to clinical translation remain. In terms of yield, current production averages about 30 µg/10⁶ MSCs, which is below the doses required for clinical periodontitis treatment. Consequently, large-scale manufacturing is essential to obtain the quantities needed for clinical use [105]. Besides, the purification process of ApoVs is complex and struggles to guarantee high purity, compounded by the heterogeneity stemming from different cell sources, making standardization particularly challenging [275]. These issues directly hamper the clinical application and dissemination of MSC-ApoVs, making effective strategies to address them urgently necessary. Therefore, systematically advancing invitro studies, pre-clinical trials, and early clinical investigations is critical for laying the translational groundwork of MSC-ApoVs in periodontitis therapy.

## Future directions

The clinical translation of MSC-ApoVs for periodontitis demands systematic investigation and optimization in six areas: biodistribution, dosing strategy, pharmacokinetics/pharmacodynamics, scalable manufacturing, safety assessment, and GMP-compliant production with quality control. First, quantitative fluorescence or radiotracer studies must determine MSC-ApoVs’ accumulation and clearance in local periodontal sites and distant organs. Second, doseescalation studies in periodontitis animal models should define the minimum effective dose, maximum tolerated dose, and optimal dosing frequency and route. Third, pharmacokinetics/pharmacodynamics analyses are required to obtain half-life, peak concentration, and targettissue exposure. Fourth, a scalable workflow is needed, pairing bioreactor-driven apoptosis induction with high-throughput purification, to deliver milligram quantities for clinical use. Fifth, long-term safety and immunogenicity should be evaluated under a chronictoxicity framework, monitoring cytokine profiles and histopathology of major organs. Sixth, a GMP pipeline should be instituted, standardizing apoptosis induction and vesicle isolation, harmonizing ultracentrifugation and FACS parameters, and testing each batch for size distribution and key surface markers to guarantee consistency.

Moreover, systematic head-to-head comparisons and in-depth mechanistic studies are essential to clarify the relative advantages of MSC-ApoVs and to inform personalized precision therapy. To date, no study has directly compared MSC-ApoVs with MSCs, their canonical exosomes, or first-line periodontal drugs within a single standardized periodontitis model; this gap underscores the need for systematic comparative trials. At the same time, data on the active components and molecular mechanisms of ApoVs in periodontitis therapy are scarce, creating an urgent need for high-throughput proteomic analysis and functional validation studies to systematically identify key effectors and pathways.

Personalized precision therapy is the next frontier. ApoVs can be customized to match individual pathological profiles by adjusting the parentcell source, preconditioning regimen, 2D/3D culture system, apoptosisinduction protocol, and isolation technique. With advances in genomics and bioinformatics, periodontitis therapy is expected to shift from conventional symptom-based classification toward genotype-based precision strategies [276, 277]. Against this backdrop, investigators can engineer ApoVs with defined protein and RNA signatures to fine-tune key gene expression and, by selecting bioactive carriers that match specific periodontal defects, achieve maximal efficacy and personalization. Through such multidimensional research and technological advances, MSC-ApoVs are poised to emerge as a precise and efficient cell-free therapy for periodontitis.

## Conclusion

In the field of periodontitis treatment, traditional methods have limited effectiveness in restoring damaged periodontal tissues, necessitating the exploration of new treatment strategies. Recently, MSCs’ transplantation therapy has made progress, but significant apoptosis of exogenous MSCs shortly after transplantation complicates their specific mechanisms of action in periodontitis treatment. Research has found that apoptotic cells can exert therapeutic effects through ApoVs, and MSC-ApoVs have shown significant therapeutic effects in various disease models, including immune modulation, inflammation inhibition, tissue regeneration, and drug delivery. This review explored the research progress and application prospects of MSC-ApoVs in the treatment of periodontitis. MSC-ApoVs can alleviate inflammation by regulating the functions of various immune cells, reducing the release of pro-inflammatory factors, and increasing the secretion of anti-inflammatory factors. Additionally, MSC-ApoVs support periodontal tissue regeneration by regulating bone metabolism and fibroblast function. As a drug delivery system, MSC-ApoVs can encapsulate specific therapeutic molecules to achieve targeted drug delivery, optimize therapeutic effects, and reduce side effects. They show potential clinical applications when combined with various scaffold structures. In summary, MSC-ApoVs show extensive application potential in the treatment of periodontitis. Their unique immune modulation, tissue regeneration, and drug delivery functions provide new research directions and clinical application references for periodontitis treatment.

## Data Availability

Not applicable.

## Abbreviations

MSCs: Mesenchymal stem cells
ApoVs: Apoptotic vesicles
LPS: Lipopolysaccharide
SRP: Scaling and root planing
ROS: Reactive oxygen species
PMNs: Polymorphonuclear neutrophils
BMSCs: Bone marrow-derived mesenchymal stem cells
PDLSCs: Periodontal ligament stem cells
DPSCs: Dental pulp stem cells
DFSCs: Dental follicle stem cells
EVs: Extracellular vesicles
ApoBDs: Apoptotic bodies
ApoMVs: Apoptotic microvesicles
ApoExos: Apoptotic exosomes-like vesicles
NETs: Neutrophil extracellular traps
NK: Natural killer
DCs: Dendritic cells
IL: Interleukin
OVX: Ovariectomy
ECs: Endothelial cells
Cu-CDs: Copper-doped carbon dots
ECM: Extracellular matrix
GTR: Guided tissue regeneration
CFUs: Colony forming units
PS: Phosphatidylserine
PC: Polycarbonate
